# Carbon Dioxide Enrichment PEBAX/MOF Composite Membrane for CO_2_ Separation

**DOI:** 10.3390/membranes11060404

**Published:** 2021-05-28

**Authors:** Po-Hsiang Tang, Pamela Berilyn So, Wa-Hua Li, Zi-You Hui, Chien-Chieh Hu, Chia-Her Lin

**Affiliations:** 1 Department of Chemistry, National Taiwan Normal University, Wenshan District, Taipei 11677, Taiwan; fetivear@gmail.com; 2 Department of Chemistry, Chung Yuan Christian University, Zhongli District, Taoyuan City 32023, Taiwan; pbtiuso@gmail.com (P.B.S.); s651724@gmail.com (W.-H.L.); zgg7674@gmail.com (Z.-Y.H.); 3 Graduate Institute of Applied Science and Technology, National Taiwan University of Science and Technology, Taipei 10607, Taiwan; 4 R&D Center for Membrane Technology, Chung Yuan Christian University, Zhongli District, Taoyuan City 32023, Taiwan

**Keywords:** metal-organic framework, carbon dioxide capture, mixed matrix membranes, MOF-polymer composite

## Abstract

Zeolitic imidazole framework (ZIF-8) was incorporated into poly(ether-block-amide) (Pebax-1657) in differing ratios to prepare mixed matrix membranes (MMMs) for gas separation. As ZIF-8 loading is increased, gas separation selectivity also gradually increases. For economic considerations, the proportion of the increase in selectivity to the amount of MOF loaded per unit was calculated. The results show that mixing 5% MOF gives the best unit performance. With this, a variety of MOFs (UiO-66, UiO-66-NH_2_, A520, MIL-68(Al) and MIL-100(Fe)) were mixed with PEBAX at 5 loading to prepare MMMs. In this work, metal-organic frameworks (MOFs) were processed using the dry-free method, where in the synthesized MOF was not dried prior to incorporation. The gas separation performance test carried out shows the highest separation performance was exhibited by P-UiO-66, wherein the CO_2_/N_2_ gas selectivity was 85.94, and the permeability was 189.77 (Barrer), which was higher than Robeson’s Upper bound in 2008, and obtained a high permeability and selectivity among mixed matrix membranes. In the preparation of high quality MMMs for gas separation, details regarding the interface phenomenon were assessed.

## 1. Introduction

In recent years, the concentration of carbon dioxide in the atmosphere has been increasing, leading to global warming and climate change. Separating carbon dioxide from emission sources such as power plants and chemical industries is an important task to combat global warming [1,2,3]. In the past few years, studies on the development of membrane technology for CO_2_/N_2_ separation and the process of capturing and storing carbon dioxide has been on the rise [4].

Compared with other porous materials, metal-organic frameworks (MOFs) possess many advantages including higher specific surface area, simpler preparation method, tunability, low cost, adjustable pore size and properties, as well as modifiable functional groups. These advantages allow us to design ideal materials for different purposes through different experimental methods. MOFs are also used in a wide variety of applications including gas separation [5,6], sensing [7], gas adsorption [8,9], selective catalysis [10,11], ion exchange [12], ultra-high purity separation [13], protein hydrolysis [14], as well as in biological conductive materials, photoelectric materials, new semiconductor materials, magnetic materials and in the field of chip development [15,16,17,18,19]. With this vast array of possibilities, high expectations have been laid on MOFs and its composite materials.

Poly(ether-block-amide) resin is best known under the trademark Pebax. Among them, Pebax^®^ MH 1657, a thermoplastic elastomer made of flexible polyether and rigid polyamide, is a promising material showing good performance in gas separation. Rigid polyamides have attracted much attention in the preparation of gas separation membrane due to its vitrification and selective properties [20,21,22,23]. In contrast, polyethers are rubbery polymers with large free volumes due to the existence of voids between their flexible chains. Since the chain activity of ether bonds is greater than that of amide bonds and can provide better permeability, in this study, Pebax-1657, which is composed of 40% amide groups and 60% ether bonds, was selected as the modified base polymer for film preparation.

Mixed matrix membranes combine the advantages of organic polymer membrane and inorganic membrane to form a new film with both characteristics. Both organic polymer films and inorganic films have the trade-off phenomenon, with high permeability and low selectivity, or low permeability and high selectivity. Therefore, preparation of MMMs aims to capture the high permeability of organic polymer membrane and the high selectivity of inorganic membrane. Addition of inorganic fillers (clay, glass, carbon black) to the polymer can reduce costs. Generally, the performance of inorganic materials depends on the degree of dispersion. Inorganic materials can be dispersed to the micron level (μm) by ultrasonication or other methods. If the dispersion can reach the nanometer level (nm), the inorganic materials will have a larger surface area to interact with polymers, and their performance will be much better than traditional composite materials. Therefore, adding inorganic materials possessing large surface areas (MOFs, zeolites, carbon molecular sieves, etc.) [24,25,26] to organic polymers can increase membrane selectivity and maintain the original membrane permeation flux. However, there are still problems that need to be resolved in the actual setting, such as the dispersion compatibility of the organic phase and the inorganic phase. It is often mentioned in the literature that MOF itself has a large specific surface area, high adsorption capacity, modifiable chemical structure, and good affinity with polymers. Therefore, researchers commonly incorporate MOFs to polymers to increase the efficiency of the film, breaking the traditional trade-off phenomenon. The Koros group has previously studied the addition of ZIF-8 to a polymer to prepare MMMs [27,28]. The results show that the film prepared by adding inorganic materials can indeed improve the performance of the film itself, with both the permeation flux and selectivity significantly improved. However, there are still some problems that need to be solved such as when the amount of the added MOF is too large, agglomerations takes place causing film defects. To address this problem, researchers use cross-linking or chemical modification to increase the adhesion between inorganic materials and polymers to prepare complete and defect-free films [29,30].

ZIF-8 is favored by many scientific researchers due to its convenient synthesis method and stability. With this said, a lot of developments can be seen with this MOF for a wide variety of applications. Its pore window is 3.4 Å, which is just between the aerodynamic diameters of N_2_ (3.6 Å) and CO_2_ (3.3 Å) so it is very suitable for this type of separation applications [31,32,33]. With the easy and convenient synthesis of ZIF-8, defects in its formation cannot be disregarded. In industrial applications, water stability is an important reference index and given that the water stability of ZIF-8 is not quite good, it is necessary to use other MOFs with better water stability to evaluate whether these MOFs may be good candidates for the preparation of MMMS. Various water stable MOFs with varying pore windows including UiO-66, UiO-66-NH_2_, MIL-68(Al), MIL-100(Fe) and A520 were used for the preparation of the MMMs. These MOFs were also considered for use due to their easy and sustainable large-scale synthesis suitable for industrial application [34,35,36,37,38].

With regards to the fact that most of the synthesized MOFs are crystalline samples, it is bound to be solidified prior to subsequent applications. In this paper, the influence of differing material preparation and mixing ratio on the carbon dioxide selectivity and flux of the composite film were evaluated. In the first step, mixed matrix films with ZIF-8 loadings of 1 wt%, 3 wt%, 5 wt%, 8 wt%, 10 wt%, 20 wt%, and 30 wt% were prepared. In reference to other literature, it can be seen that the Pebax@MH 1657 polymer has a very good affinity for carbon dioxide. The use of this polymer to make membranes can increase the carbon dioxide permeation flux and separation efficiency. Therefore, polyether polyamide block copolymer was selected, and subsequent screening for the most suitable Pebax/MOF mixing ratio to prepare the composite film was carried out.

## 2. Materials and Methods

### 2.1. Chemicals

Zinc nitrate hexahydrate (Zn(NO_3_)_2_·6H_2_O, Showa (Gyoda, Japan), ≥99%), zirconium chloride (ZrCl_4_, Sigma-Aldrich (Saint Louis, MO, USA), ≥99%), aluminum chloride hexahydrate (AlCl_3_·6H_2_O, Alfa (Lancashire, UK), ≥98%), aluminum sulfate octahydrate (Al_2_(SO_4_)_3_·18H_2_O, J.T. Baker (Radnor, PA, USA), ≥98%), aluminum nitrate nonahydrate (Al(NO₃)₃·9H₂O, Merck (Darmstadt, Germany), ≥99%), ferrous chloride tetrahydrate (FeCl_2_·4H_2_O, Showa (Gyoda, Japan), ≥99%), sodium hydroxide (NaOH, Fluka-Honeywell (Charlotte, NC, USA), ≥99%), 2-methylimidazole (2-MeIM, Acros (Geel, Belgium), ≥99%), 1,4-benzenedicarboxylic acid (p-H_2_BDC, TCI (Tokyo, Japan), ≥98%), 2-aminoterephthalic acid (p-H_2_BDC-NH_2_, Alfa, ≥98%), fumaric acid (H_2_FUM, Alfa, ≥99%), methanol (MeOH, CH_3_OH, Merck, ≥99.5%), ethanol (EtOH, TCI, 99.5%), Pebax^@^ MH 1657 (Arkema(Colombes, France)), 2-propanol (isopropyl alcohol, IPA, C_3_H_7_OH, J.T. Baker, tech. grade, 99%), N,N-dimethylformamide (DMF, C_3_H_7_NO, Merck, 99.5%).

### 2.2. Material Synthesis 

#### 2.2.1. Synthesis of ZIF-8

Zn(NO_3_)_2_·6H_2_O (2.933 g, 9.87 mmol) and 2-MeIM (6.489 g, 79.04 mmol) were dissolved in 100 mL methanol separately. The two solutions were stirred together at room temperature and slowly became turbid. After 1 h, the nanocrystals were washed with ethanol and collected by centrifugation. The nanocrystals were stored in ethanol prior to use [39].

#### 2.2.2. Synthesis of UiO-66

ZrCl_4_ (0.54 mmol, 0.125 g) and H_2_BDC (0.75 mmol, 0.123 g) were dissolved in 30 mL DMF, then 2 mL of HCl was added. The reaction is stirred at 80 °C for 24 h. The crystals were washed twice with DMF, twice with ethanol, and collected by centrifugation. The crystals were stored in ethanol prior to use [40].

#### 2.2.3. Synthesis of UiO-66-NH_2_

Dissolve ZrCl_4_ (0.54 mmol, 0.125 g) and H_2_BDC-NH_2_(0.75 mmol, 0.136 g) were dissolved in 30 mL DMF, then 2 mL of HCl was added. The reaction is stirred at 80 °C for 24 h. The crystals were washed twice with DMF, twice with ethanol, and collected by centrifugation. The crystals were stored in ethanol prior to use [40].

#### 2.2.4. Synthesis of MIL-68(Al)

AlCl_3_·6H_2_O (2 mmol, 0.4828 g) was added to 1 mmol (0.1661 g) of H_2_BDC in 8 mL of IPA. The mixture was stirred under reflux for 48 h. The obtained white solids were washed with IPA three times, washed with ethanol four times, collected by centrifugation, and finally stored in ethanol [35].

#### 2.2.5. Synthesis of A520

Al_2_(SO_4_)_3_·18 H_2_O (0.084 mol, 55.976 g) was dissolved in 30 mL H_2_O. In a separate container, H_2_FUM (0.169 mol, 19.576 g) and NaOH (0.506 mol, 20.256 g) were dissolved in 30 mL H_2_O. The two solutions were mixed together and stirred at 60 °C for 6 h. The obtained solids were washed with ultrapure water three times, washed with ethanol, collected by centrifugation, and finally stored in ethanol [41].

#### 2.2.6. Synthesis of MIL-100(Fe)

H_3_BTC (7.6 mmol, 1.676 g) was dissolved in 1 M NaOH (23.72 g) to form solution A, while FeCl_2_·4H_2_O (11.4 mmol, 2.26 g) was dissolved in 97.2 g H_2_O to form solution B. Solution A was slowly added into solution B dropwise, and stirred at room temperature for 12 h. The solution changed from green to orange-brown. The obtained products were washed with water three times, washed with ethanol, collected by centrifugation, and then stored in ethanol [34].

### 2.3. Characterization of Synthesized MOFs

Characterization of the synthesized MOFs were carried out by taking 5 mL of the MOFs immersed in ethanol. The samples were separated from the solvent by centrifugation, and heated at 120 °C under vacuum for 24 h to obtain a dry powder sample. The powder pattern of the MOFs was measured using a Bruker D8 advance powder X-ray diffractometer (PXRD) with Cu Kα radiation (λ = 1.54056 Å) at room temperature scanning at a 2θ range from 2° to 40°. BET surface areas of the synthesized MOFs were computed by measuring the nitrogen adsorption using the Micromeritics ASAP2020 surface area and porosimetry system. Prior to analysis, the MOFs were degassed at 120 °C for 24 h.

### 2.4. Preparation of Membrane Film

Pebax was added to the solvent (CH_3_OH:H_2_O = 7:3) and stir evenly to form a 6 wt% Pebax casting solution. This was stirred continuously for 6 h at a speed of 300 rpm at 80 °C. Afterwards, 3 mL of the casting solution was poured into a 6 cm-diameter petri dish and placed in an oven at 70 °C for 2 h to have the solvent evaporated. The dried film was directly removed and denoted as “P”.

#### 2.4.1. Preparation of Pebax/ZIF-8 MMMs

ZIF-8 was added to the solvent (CH_3_OH: H_2_O = 7:3) and ultrasonicated for 30 min to make it uniformly dispersed. Pebax was subsequently added and the mixture was stirred continuously at 300 rpm at 80 °C for 6 h. After Pebax is completely dissolved, 3 mL of the casting solution was poured into a 6 cm-diameter petri dish then placed in an oven at 70 °C for 2 h to evaporate the solvent. The dried film can be taken off directly and denoted as “P-Z*x*”, where *x* is the mixing ratio of ZIF-8. The different mixing ratios that were prepared are shown in Table 1.

#### 2.4.2. Preparation of 5% Pebax/MOF MMMs

The selected MOF (0.0822 g) was added to 25.83 g of the solvent (CH_3_OH: H_2_O = 7:3), and ultrasonicated for 30 min to make it uniformly dispersed. Pebax (1.6485 g) was subsequently added and the mixture was stirred continuously at 300 rpm at 80 °C for 6 h. After complete dissolution of Pebax, 3 mL of the casting solution was poured into a 6 cm-diameter petri dish. Afterwards, it was placed in an oven at 70 °C for 2 h to have the solvent thoroughly evaporated. The dried film was directly peeled off from the substrate and is denoted as “P-MOF”, wherein MOF is the specific metal-organic framework (UiO-66, UiO-66-NH_2_, MIL-53(Al), A520, MIL-68(Al) and MIL-100(Fe)) used in the preparation of the MMM.

### 2.5. Characterization of MMMs

#### 2.5.1. Powder X-ray Diffraction (PXRD)

The powder pattern of the MMMs were measured using a Bruker D8 advance powder X-ray diffractometer (PXRD) with Cu Kα radiation (λ = 1.54056 Å) at room temperature scanning at a 2θ range from 2° to 50°. Because crystals with different structures have different atomic arrangements and different diffraction positions, the powder diffraction pattern is compared with the theoretical diffraction pattern calculated from a single crystal in the experiment. It is proved that the sample powders obtained in the experiment are all composed of the same single crystal, which is the so-called single phase or pure phase.

#### 2.5.2. Scanning Electron Microscopy (SEM)

Scanning electron microscopy (SEM) images were captured on a JEOL JSM-7600F FE-SEM (Tokyo, Japan). Sample preparation was carried out by subjecting the prepared MMM to sub-zero temperatures by immersion in liquid nitrogen, subsequently freezing and dewatering the membranes. A 5mm × 5mm membrane sample was cut and fixed onto the sample stage using double-sided copper tape. Prior to SEM imaging, the samples were then platinum-coated for 100 s to make it conductive.

#### 2.5.3. Thermogravimetric Analysis (TGA)

A DuPont TA Q50 thermogravimetric analyzer was used to conduct the experiments. Powdered samples of around 15–20 mg were placed in a clean platinum hanging pan and equilibrated. Samples were running under nitrogen gas (flow rate 40 cc/min), from 30 °C to 800 °C at a rate of 10 °C per minute with weight changes were recorded. Taking weight% as the vertical axis and temperature as the horizontal axis, the correlation curve can be obtained, and then the thermal decomposition of the compound and its thermal stability can be determined. 

#### 2.5.4. Gas Permeation Analyzer (GPA)

A Yanaco GTR-11MH Gas permeation analyzer was used to conduct the experiments. The prepared film was placed into the cell of the gas permeation test device. A vacuum pump was used to vacuum the downstream end of the cell followed by the subsequent introduction of the test gas to the upstream end. After the gas flow rate is stable and the vacuum at the downstream end reaches 10-2 torr, the valves of the downstream cell were opened. When the specified test time is reached, the valve of the downstream cell was closed then the gas collection tube of the GC was connected to analyze the volume of the gas that passed through the membrane to calculate the gas permeability. Different gases (N_2_, CO_2_, He) were tested using the same way. In addition, the ideal separation coefficient of the gases was calculated.

## 3. Results and Discussion

**Figure 1:**
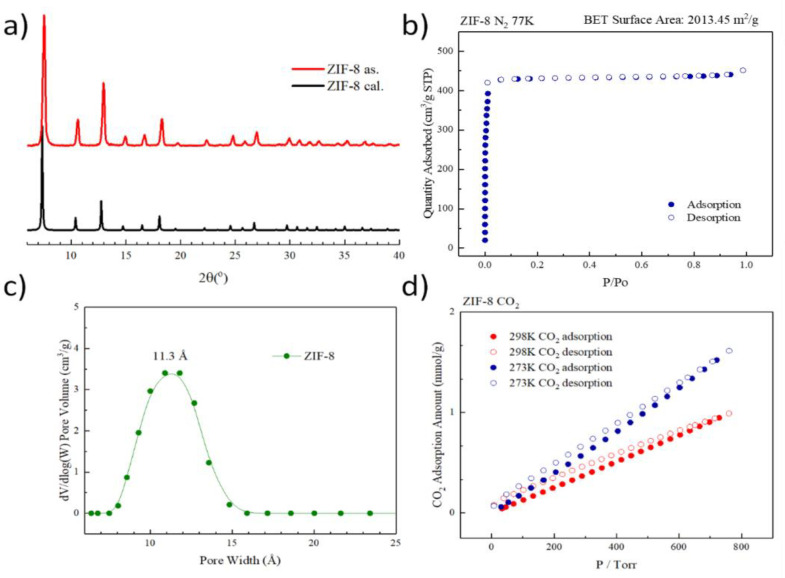
Characterization of synthesized ZIF-8. (**a**) the PXRD pattern, (**b**) N_2_ adsorption isotherm (77 K), (**c**) pore size distribution, and (**d**) CO_2_ adsorption isotherm (298/273 K).

Characterization of the synthesized ZIF-8 were carried out using a powder diffractometer where the measured pattern was comparable with the calculated data as shown in Figure 1a. The BET surface area was computed to be 2013.45 m^2^/g N_2_ adsorption measurement at 77 K (Figure 1b). Among them, the adsorption test at low partial pressure (p/po < 0.2) confirmed that ZIF-8 has a microporous, and the adsorption model of type-I also proved this, and then calculated by DFT theory The model shows that the material has an average hole size of 11.3 Å (Figure 1c), CO_2_ adsorption/desorption isotherms (Figure 1d) were also measured exhibiting a CO_2_ adsorption capacity of 0.99 mmol/g at 760 torr, 25 °C, and 1.62 mmol/g at 0 °C. The rest of the MOF characterization measurement including PXRD, N_2_ and CO_2_ adsorption isotherms, as well as thermogravimetric analyses can be seen in the supporting information (Figures S2–S22). 

**Figure 2:**
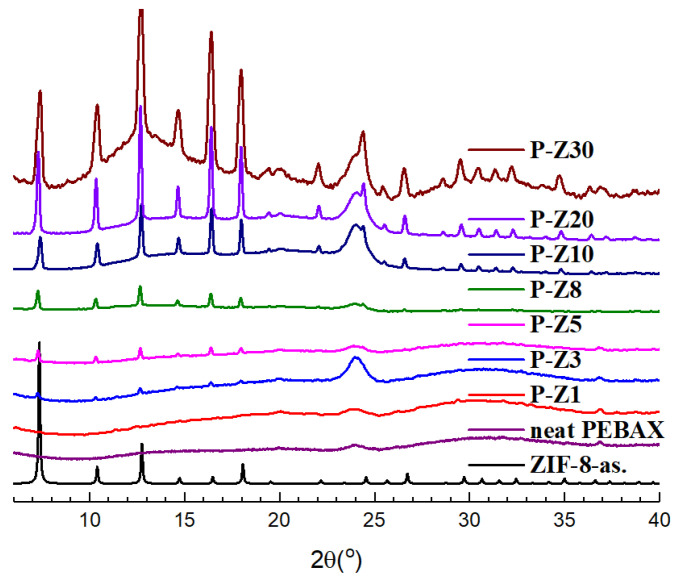
Powder pattern of Pebax/ n wt% ZIF-8 MMMs.

Upon successful synthesis of ZIF-8, it was incorporated into the Pebax at varying mixing ratios. The prepared MMMs were then subjected for PXRD measurement. As Pebax is inherently not very crystalline, a smooth pattern with a characteristic broad peak at 24° 2θ can be observed with the prepared neat Pebax membrane (Figure 2), a stark contrast with the PXRD pattern of ZIF-8 which is highly crystalline in nature. With the first MMM prepared, P-Z1, it can be observed that the peaks for ZIF-8 is not discernable as the amount of ZIF-8 incorporated is very low, and the particles are widely distributed throughout the membrane. As shown in Figure 2, as the mixing ratio for ZIF-8 is increased, the characteristic peaks of ZIF-8 become more evident proving the successful incorporation of ZIF-8 into the MMMs.

Characterization of the MOF samples were carried out by comparison of their PXRD pattern with the theoretical or calculated PXRD patterns of the said MOFs as shown in Figure S1. The characteristic peaks in the diffraction pattern of the synthesized MOFs coincides with that of the calculated diffraction patterns, proving that the syntheses were successful. As observed in Figure S23, it can be seen that upon incorporation of the MOFs with Pebax to prepare the MMMs, the MOFs still retain their inherent crystallinity as exhibited by the characteristic peaks of the MOFs in the measured PXRD patterns. 

**Figure 3:**
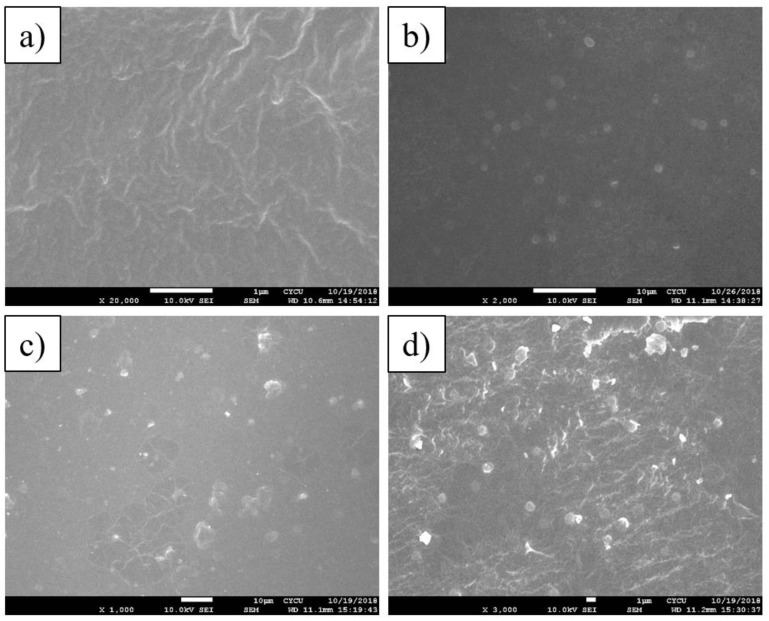
SEM image for P-Z5(dry-free) [(**a**): surface, (**b**): cross-section] and P-Z5(Dried) [(**c**): surface, (**d**): cross-section].

From Figure 3, it can be observed that P-Z5 (dry-free) exhibits better dispersion without the presence of MOF particle agglomeration, as opposed to that of P-Z5 (dried) wherein particle agglomeration is evident. With this, it can be deduced that the preparation of MMM without the first drying the MOFs proved to be better than the conventional methods of drying the MOF prior to MMM incorporation. In Figures S24–S31, it can be observed that the number of ZIF particles from P-Z1 to P-Z30, increased significantly, as expected when the amount of ZIF-8 incorporated is increased.

**Figure 4:**
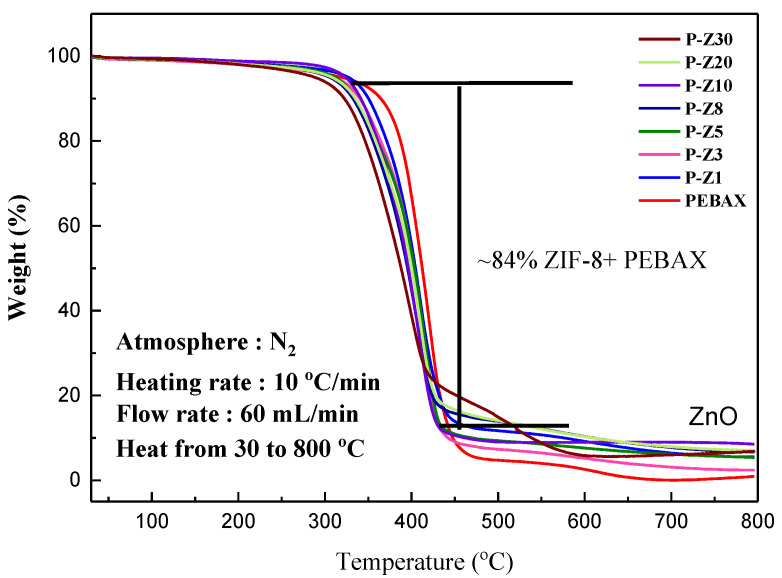
TGA curve of Pebax/n wt% ZIF-8 MMM.

It can be observed from TGA that when the loading of ZIF-8 in the mixed matrix membrane increases, its thermal stability will gradually decrease (Figure 4). This is due to the fact that the increase of ZIF-8 will reduce the mechanical properties of the Pebax membrane. Initially, without MOF incorporation, Pebax is stable until 300 °C. This value was reduced to 200 °C with the addition of ZIF-8. 

Comparison of the MMMs generated by the incorporation of 5% MOF by their TGA curves shows that a weight loss of nearly 80% starting at 300 °C was observed for the ZIF-8 MMM, while in P-MIL-68(Al) and P-MIL-100(Fe) has a second stage weight loss at 450 °C because MIL-68 (Al) and MIL-100 (Fe) have higher thermal stability than other MOFs, and even more thermally stable than Pebax (Figure S32).

**Figure 5:**
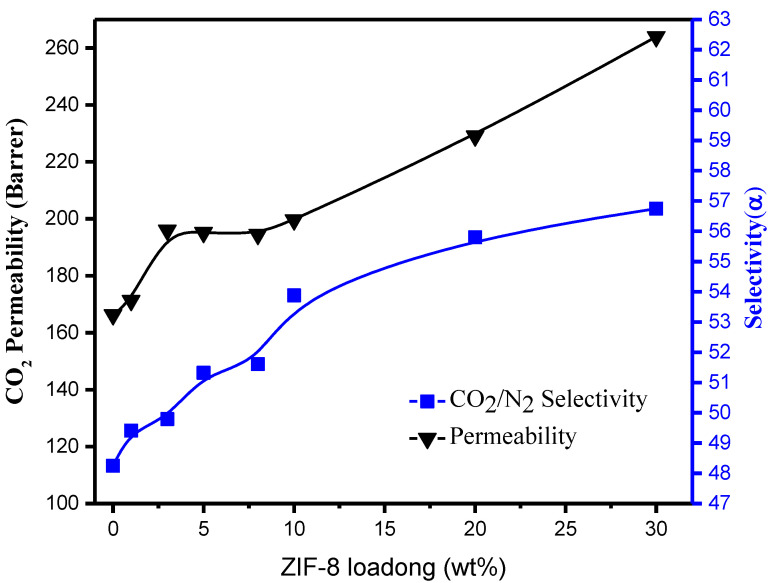
Pebax/n wt% ZIF-8 MMMs gas permeability and gas separation selectivity performance.

**Figure 6:**
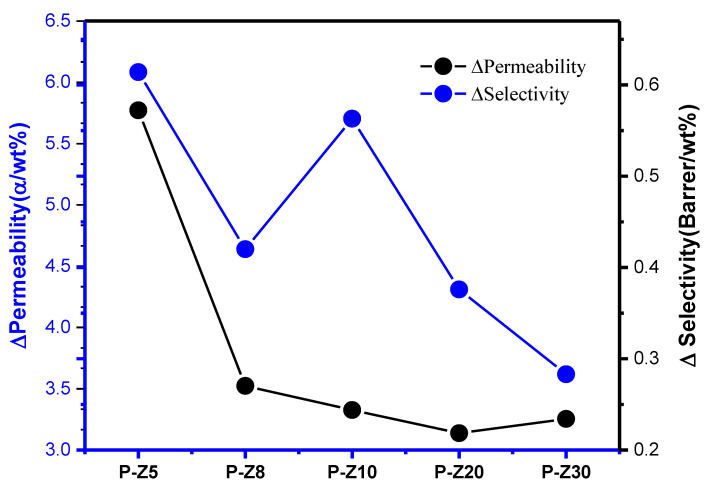
Pebax/n wt% ZIF-8 MMMs can improve the performance ratio per wt%.

A gradual increase in gas selectivity of the Pebax/ZIF-8 MMMs produced by the dry-free method was observed after the ZIF-8 loading is increased, and compared with other MMMs produced using ZIF-8, the current samples prepared exhibited better results (Figure 5). The gas selectivity of 10 wt% ZIF-8 is increased by about 10%; the selectivity of 20 wt% ZIF-8 is increased by about 17%, while the selectivity of 30 wt% ZIF-8 is increased by about 20% (Figure S33). Comparing the efficiency ratio improvement per wt% MOF, it was found that adding 5% MOF has the highest efficiency ratio, so this mixing ratio was used for other MOFs to prepare the MMMs (Figure 6).

**Figure 7:**
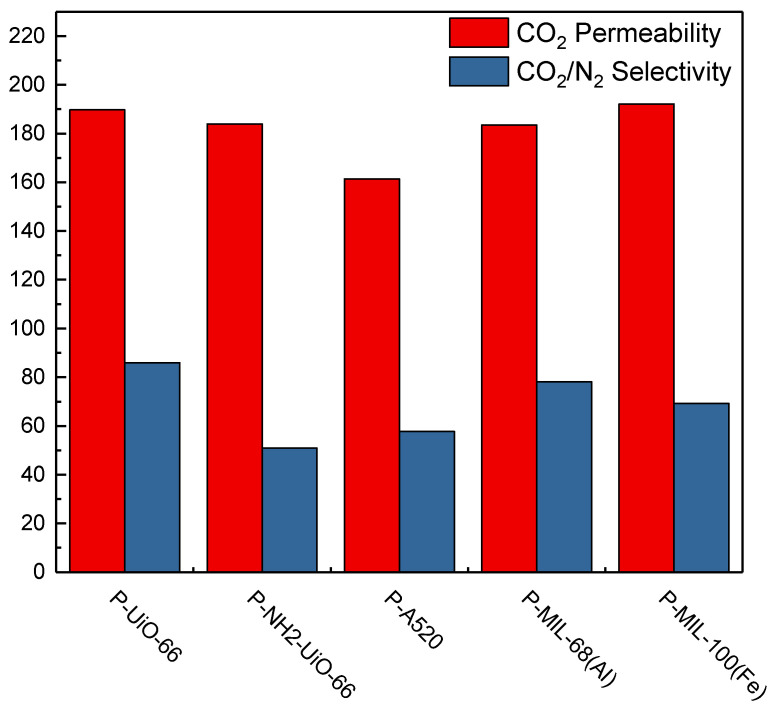
Gas separation efficiency diagram of PEBAX/5wt% MOF.

**Figure 8:**
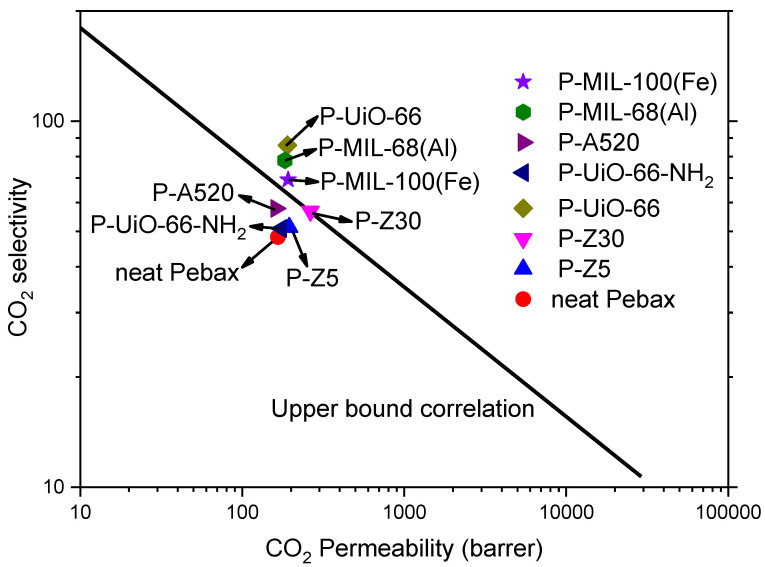
Upper bound correlation (2008).

The gas separation test results of the prepared MMM is presented in Figure 7. With reference to their gas separation selectivity, P-UiO-66, exhibited the highest selectivity followed by P-MIL-100(Fe), P-ZIF-8, P-MIL-68(Al), P-UiO-66-NH_2_, with P-A520 showing the lowest selectivity. Analyzing the reasons for arrangement, MIL-68(Al) and A520 are all 1-D chains, while UiO-66, UiO-66-NH_2_, MIL-100(Fe), and ZIF-8 are 3-D chains. A 1-D chain structure has only one channel for gas to pass through, and the direction of the channel cannot be controlled, so it is less efficient than the other MOFs. The reason for the poorest separation performance of P-A520 is the use of rapid synthesis. The crystallinity of A520 is not good as observed from PXRD, and the structure is incomplete. It can also be seen from the DFT pore size calculated by N_2_ 77 K adsorption and desorption that there are too many irregular pores, which shows that A520 has many defects and reduces its selectivity. Due to its 1-D channel, MIL-68 (Al) has fewer pores for gas penetration as compared to other 3-D channel MOFs, which in turn affects gas selectivity. UiO-66-NH_2_ was also synthesized rapidly and it can be seen from PXRD that its structure is not completely constructed and has defects. Because it has the amino (NH_2_) functional groups while Pebax has hydroxyl (OH) groups, the interaction generates hydrogen bonds, which leads to increased CO_2_ circulation resistance. UiO-66, which has the best separation ability, has 3-D channels and multiple channels to allow gas to circulate, and UiO-66 has strong water resistance. Even if it is mixed with a solvent, it still retains its good crystallinity. Robeson sorted out a lot of polymer gas membrane separation performance data to make maps, and proposed an Upper bound in 1991 and 2008 respectively, wherein CO_2_/N_2_ separation was selected for comparison. The excellent CO_2_/N_2_ separation results of P-UiO-66 in comparison to other materials is shown in Figure 8 (Tables S1, S2 and S4). 

## 4. Conclusions

In this paper, we conclude that the dry-free processing method of MOFs was done to successfully prepare the Pebax/MOF MMM, which reduced the void volume of the interface between the MOF and the polymer, and improved the gas separation efficiency of carbon dioxide and nitrogen. In addition, the UiO-66 material is the best choice for the Pebax/MOF mixed matrix film. Because of the conditions that do not require heat treatment and the advantages of its 3-D channel, the CO_2_/N_2_ gas separation of the P-UiO-66 mixed matrix film displayed the highest performance. The gas permeation flux reaches 189.77 Barrer, and the gas separation selectivity is as high as 85.94, surpassing the Upper bound proposed by Robeson in 2008, giving this membrane a potential for commercialization.

## Figures and Tables

**Table 1:** Metal-organic framework (MOF) ratios for preparation of casting solution.

Samples	PEBAX	Solvent (CH_3_OH:H_2_O = 7:3)	ZIF-8
P	3.297 g	51.65 g (36.16 g + 15.50 g)	X
P-Z1	1.6485 g	25.83 g (18.08 g + 7.75 g)	0.0164 g
P-Z3	1.6485g	25.83 g (18.08 g + 7.75 g)	0.0493 g
P-Z5	1.6485 g	25.83 g (18.08 g + 7.75 g)	0.0822 g
P-Z8	1.6485 g	25.83 g (18.08 g + 7.75 g)	0.1316 g
P-Z10	1.6485 g	25.83 g (18.08 g + 7.75 g)	0.1645 g
P-Z20	1.6485 g	25.83 g (18.08 g + 7.75 g)	0.3290 g
P-Z30	1.6485 g	25.83 g (18.08 g + 7.75 g)	0.4935 g

## Data Availability

Not applicable.

## Supplementary Materials

The following are available online at https://www.mdpi.com/article/10.3390/membranes11060404/s1, Figure S1: Comparison of PXRD pattern of the synthesized MOFs with the calculated PXRD patterns, Figure S2: UiO-66 N_2_ isotherm, Figure S3: UiO-66 pore size distribution, Figure S4: UiO-66 CO_2_ isotherm, Figure S5: UiO-66-NH_2_ N_2_ isotherm, Figure S6: UiO-66-NH_2_ pore size distribution, Figure S7: UiO-66-NH_2_ CO_2_ isotherm, Figure S8: MIL-68(Al) N_2_ isotherm, Figure S9: MIL-68(Al) pore size distribution, Figure S10: MIL-68(Al) CO_2_ isotherm, Figure S11: MIL-100(Fe) N_2_ isotherm, Figure S12: MIL-100(Fe) pore size distribution, Figure S13: MIL-100(Fe) CO_2_ isotherm, Figure S14: A520 N_2_ isotherm, Figure S15: A520 pore size distribution, Figure S16: A520 CO_2_ isotherm, Figure S17: TGA curve of ZIF-8, Figure S18: TGA curve of UiO-66, Figure S19: TGA curve of UiO-66-NH_2,_ Figure S20: TGA curve of A520, Figure S21: TGA curve of MIL-68(Al), Figure S22: TGA curve of MIL-100(Fe), Figure S23: PXRD pattern of (a) P-UiO-66, (b) P-UiO-66-NH_2_, (c) P-A520 (d) P-MIL-68(Al), (e) P-MIL-100(Fe), Figure S24: Neat PEBAX SEM image, Figure S25: P-Z1 SEM image, Figure S26: P-Z3 SEM image, Figure S27: P-Z5 SEM image, Figure S28: P-Z8 image, Figure S29: P-Z10 image, Figure S30: P-Z20 image, Figure S31: P-Z30 image, Figure S32: TGA curve of Pebax/5 wt% MOF MMMs and neat Pebax, Table S1: N_2_ Adsorption, Table S2: CO_2_ Adsorption, Table S3: PEBAX/n wt% ZIF-8 MMMs gas separation efficiency, Table S4: PEBAX/5wt% MOF GPA measurement, Figure S33: The gas permeability and gas separation selectivity of Pebax/ZIF-8 MMMs in the literature.
