# Supplementary material for: Carbon Dioxide Enrichment PEBAX/MOF Composite Membrane for CO2 Separation
Source: Membranes (Basel). 2021 May 28;11(6):404. doi: 10.3390/membranes11060404 (PMC8228013; doi:10.3390/membranes11060404)
Supplement: Supplementary file 1 [file membranes-11-00404-s001.zip › membranes-1225999-supplementary.pdf]

Supporting Information

Carbon Dioxide Enrichment PEBAX/MOF Composite Membrane for CO_2 Separation

Po-Hsiang Tang [1], Pamela Berilyn So [2], Wa-Hua Li [2], Zi-You Hui [2], Chien-Chieh Hu [3,4,*] and Chia-Her Lin [1,4,*]

[1] Department of Chemistry, National Taiwan Normal University, Wenshan District, Taipei 11677, Taiwan; fetivear@gmail.com

[2] Department of Chemistry, Chung Yuan Christian University, Zhongli District, Taoyuan City 32023, Taiwan; pbtiuso@gmail.com (P.B.S.); s651724@gmail.com (W.-H.L.); zgg7674@gmail.com (Z.-Y.H.)

[3] Graduate Institute of Applied Science and Technology, National Taiwan University of Science and Technology, Taipei 10607, Taiwan

[4] R&D Center for Membrane Technology, Chung Yuan Christian University, Zhongli District,
Taoyuan City 32023, Taiwan

* Correspondence: cchu@mail.ntust.edu.tw (C.-C.H.); chiaher@ntnu.edu.tw (C.-H.L.)

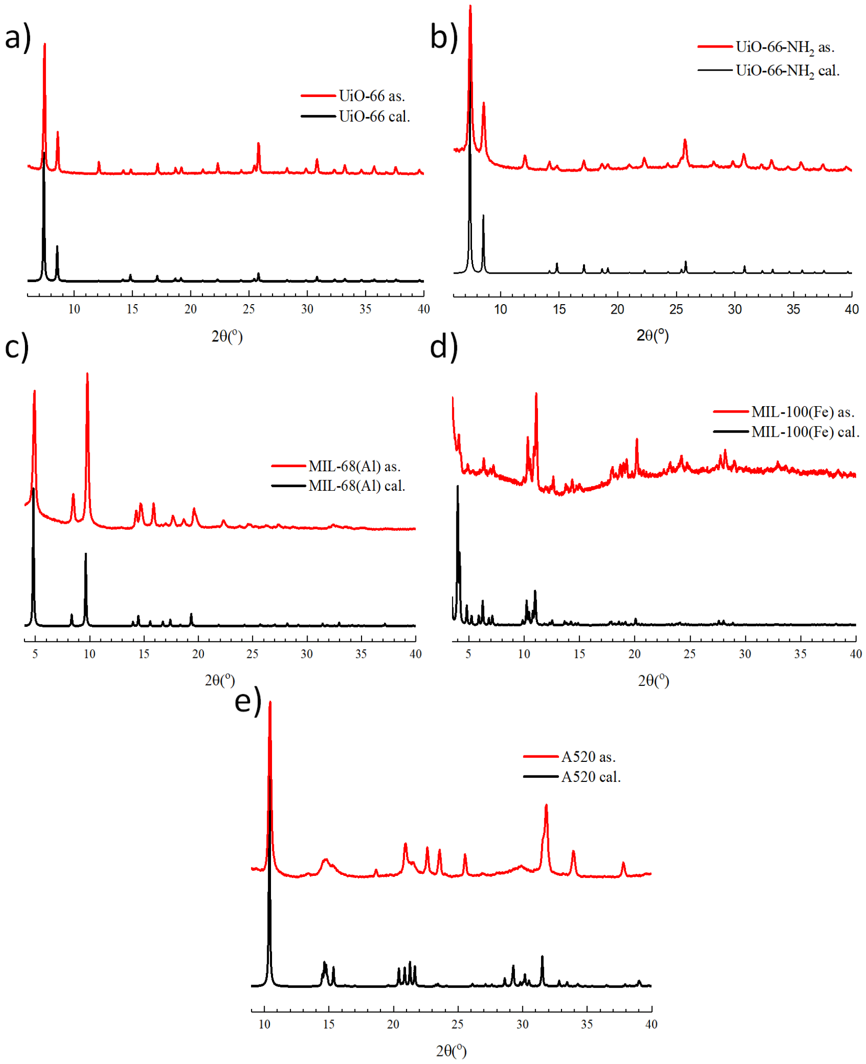

Figure S1. Comparison of PXRD pattern of the synthesized MOFs with the calculated PXRD patterns. a) ZIF-8, b) UiO-66, c) UiO-66-NH_2, d) MIL-68(Al), e) MIL-100(Fe), f) A520.

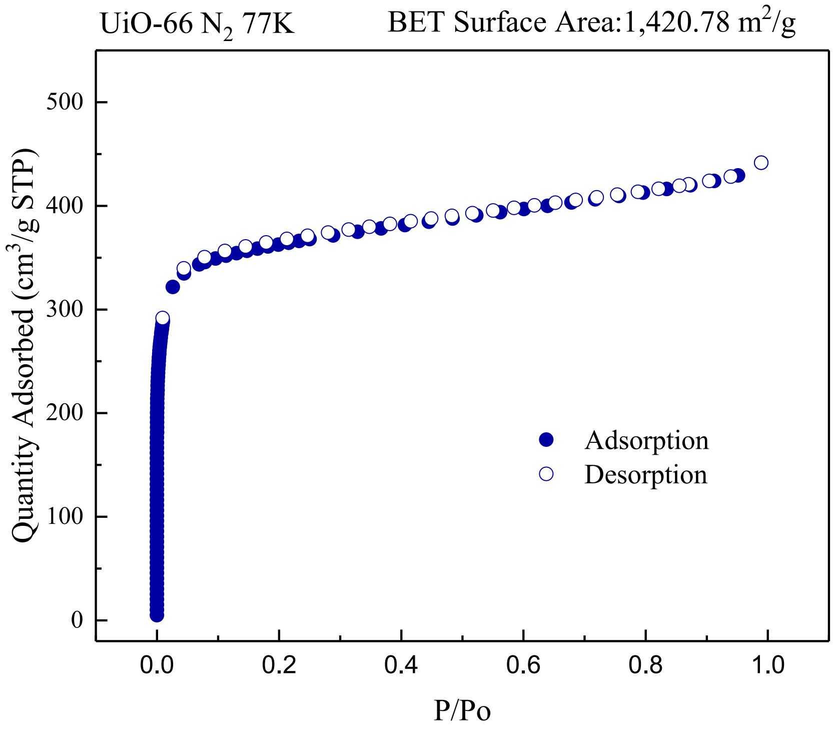

Figure S2. UiO-66 N_2 isotherm.

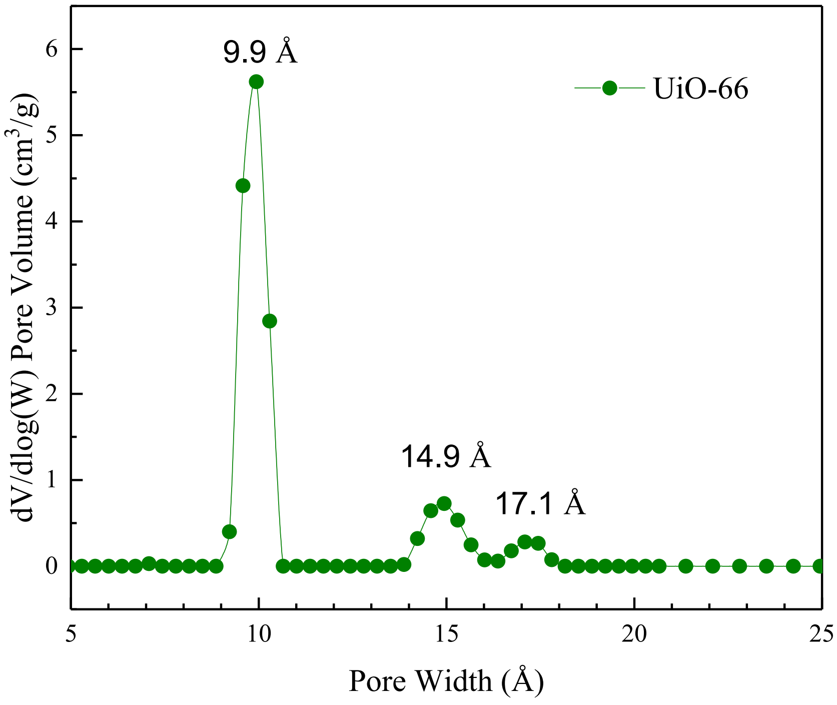

Figure S3. UiO-66 pore size distribution.

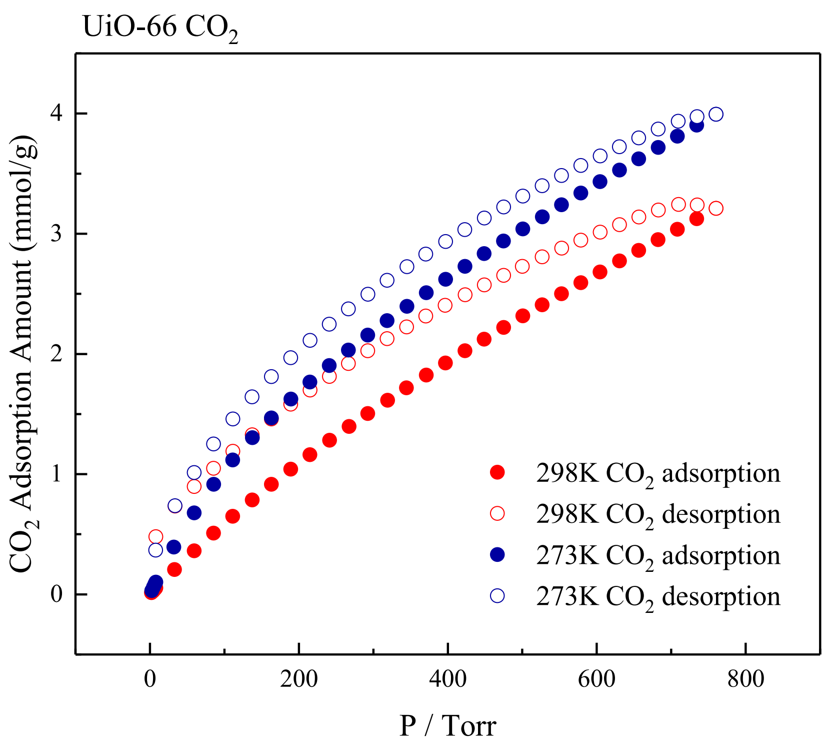

Figure S4. UiO-66 CO_2 isotherm.

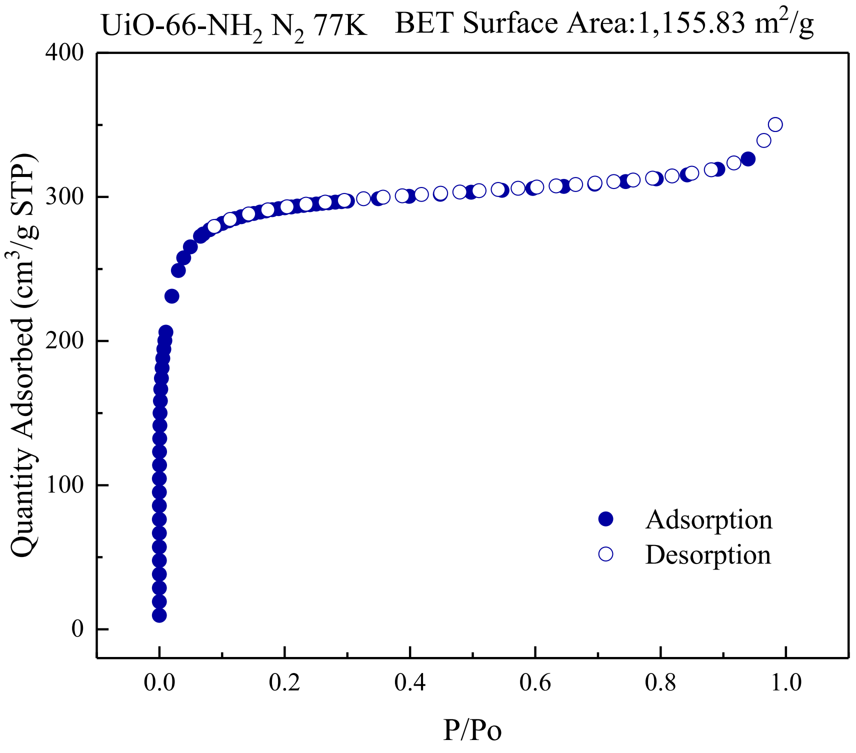

Figure S5. UiO-66-NH_2 N_2 isotherm.

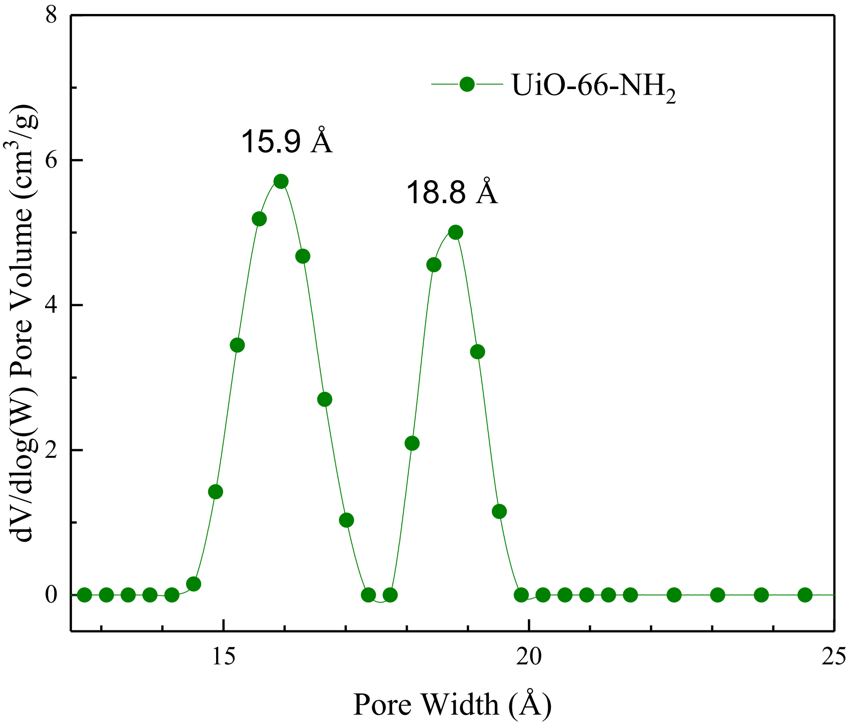

Figure S6. UiO-66-NH_2 pore size distribution.

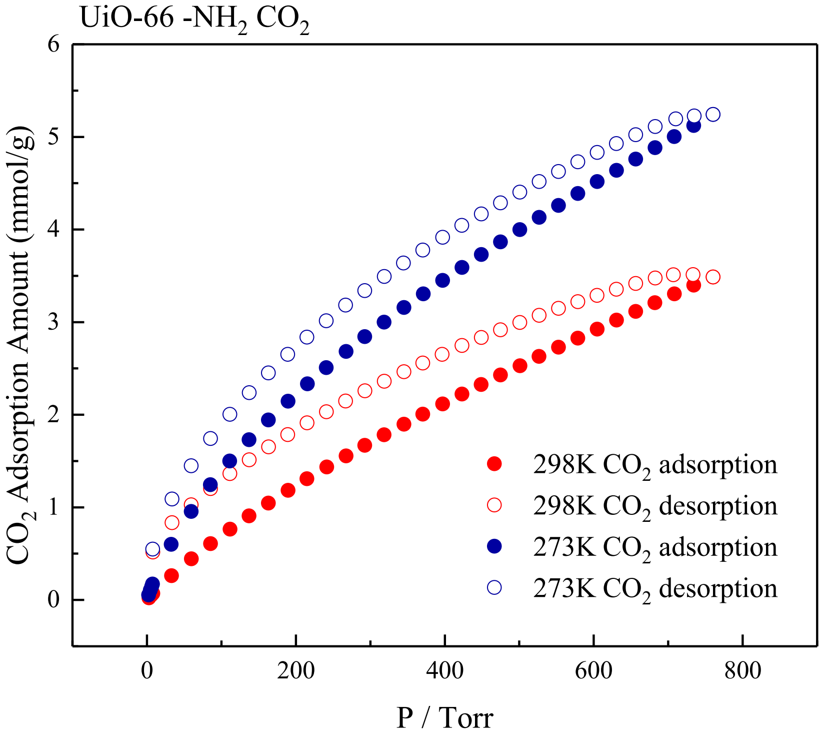

Figure S7. UiO-66-NH_2 CO_2 isotherm.

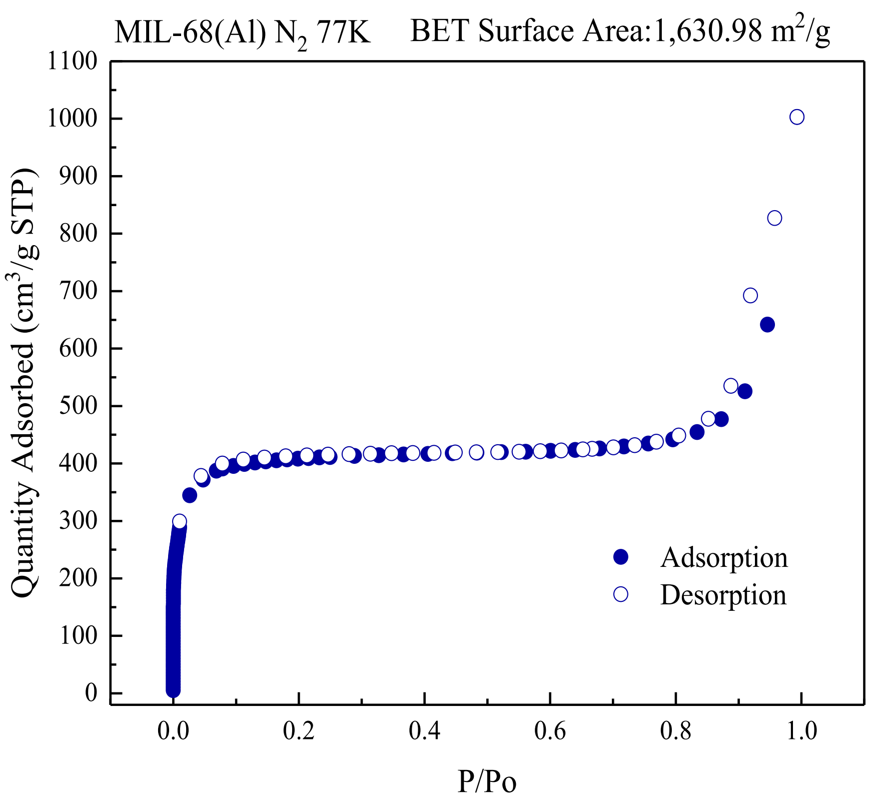

Figure S8. MIL-68(Al) N_2 isotherm.

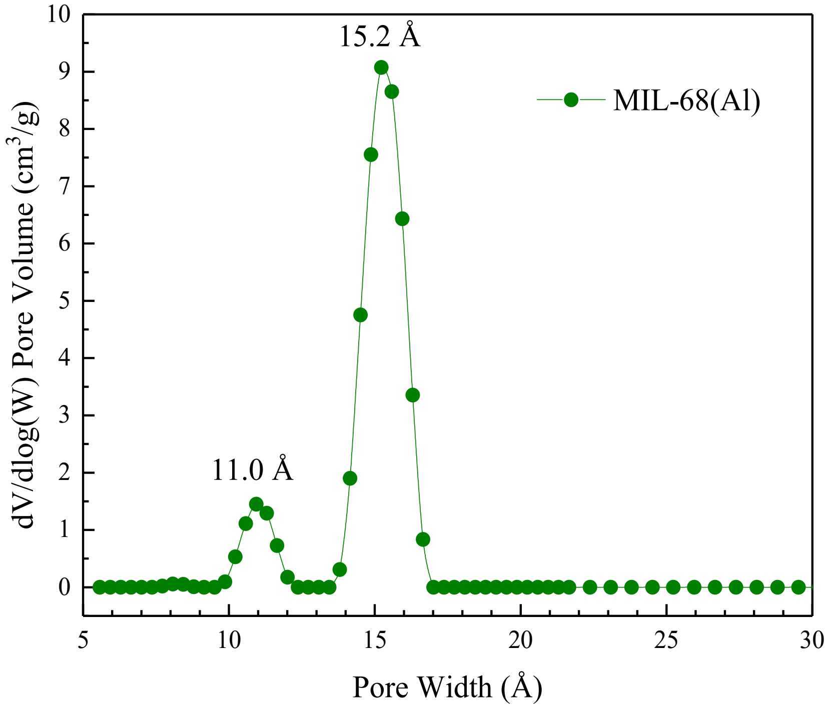

Figure S9. MIL-68(Al) pore size distribution.

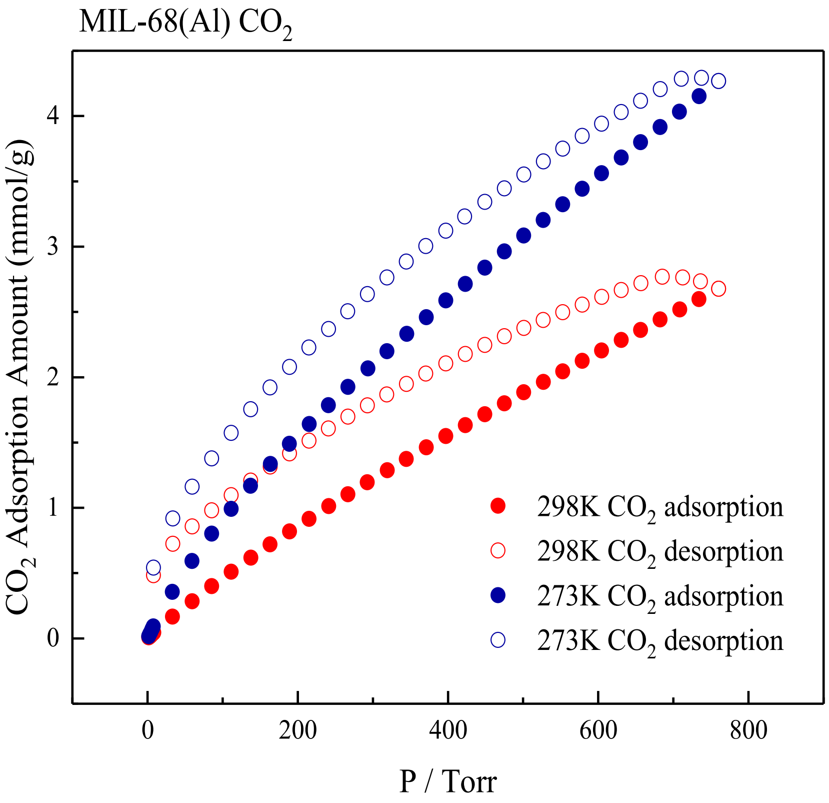

Figure S10. MIL-68(Al) CO_2 isotherm.

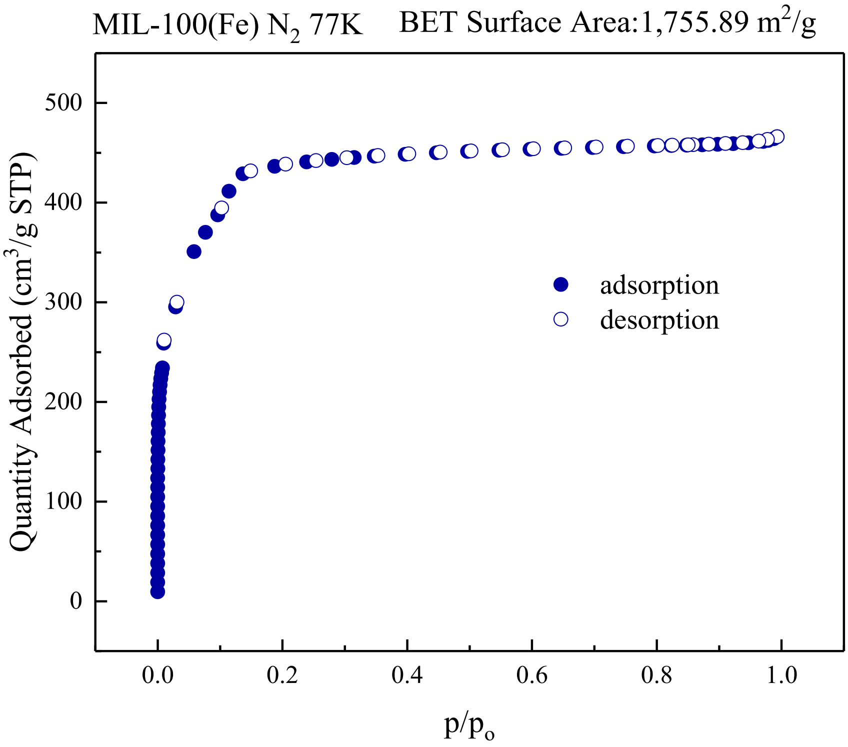

Figure S11. MIL-100(Fe) N_2 isotherm.

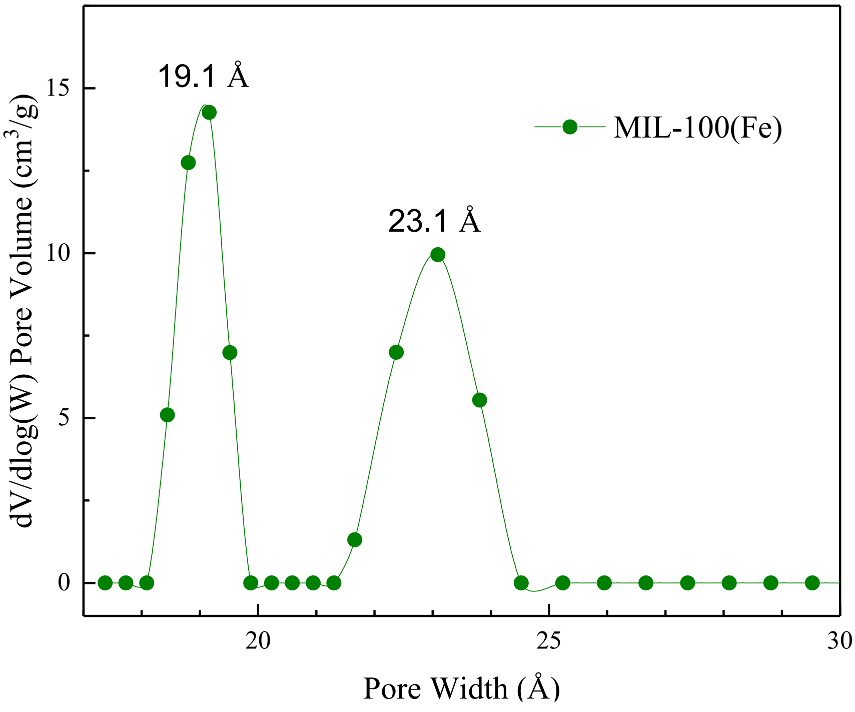

Figure S12. MIL-100(Fe) pore size distribution.

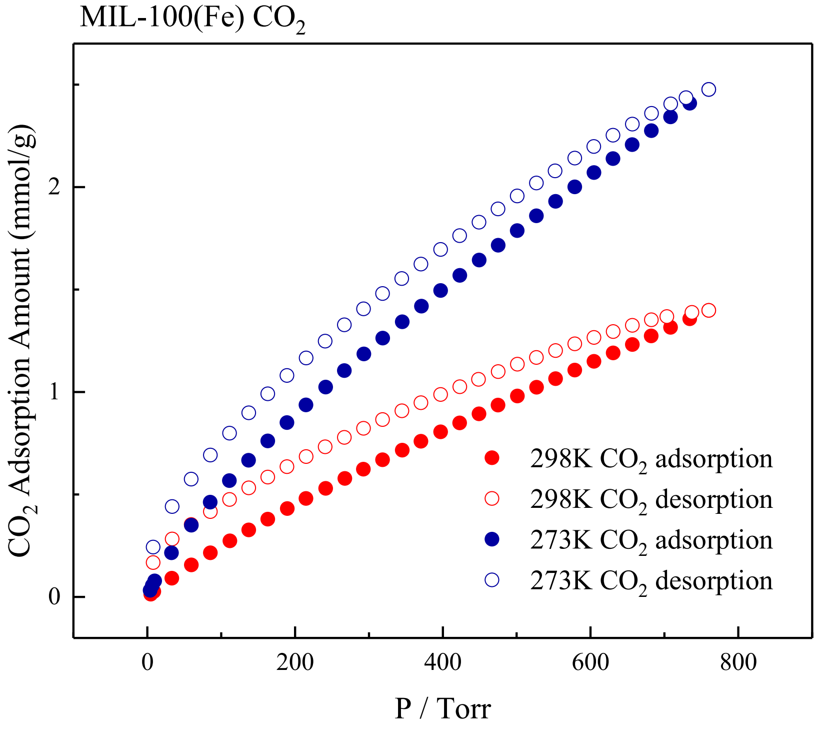

Figure S13. MIL-100(Fe) CO_2 isotherm.

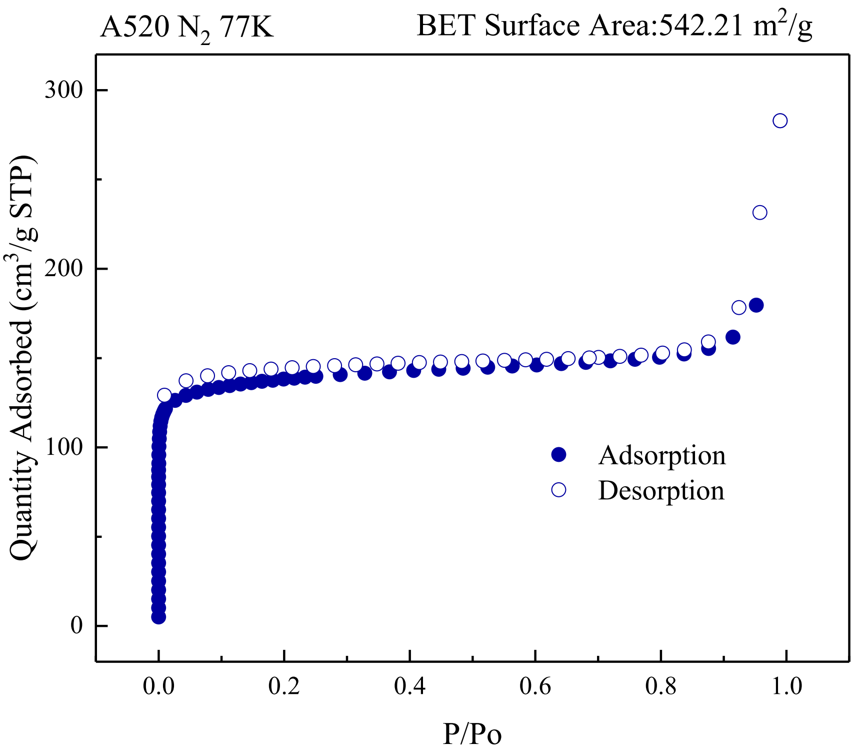

Figure S14. A520 N_2 isotherm.

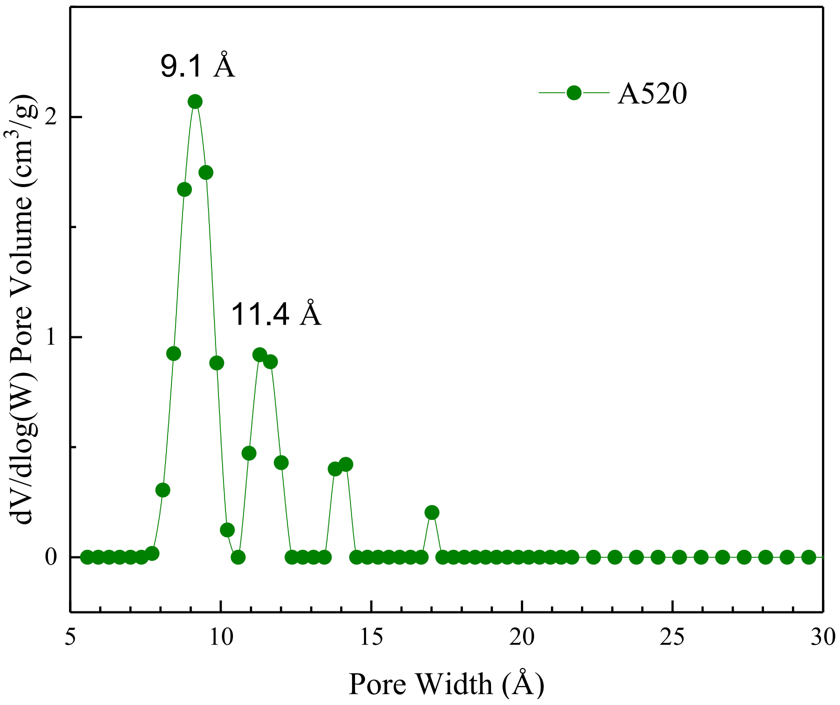

Figure S15. A520 pore size distribution.

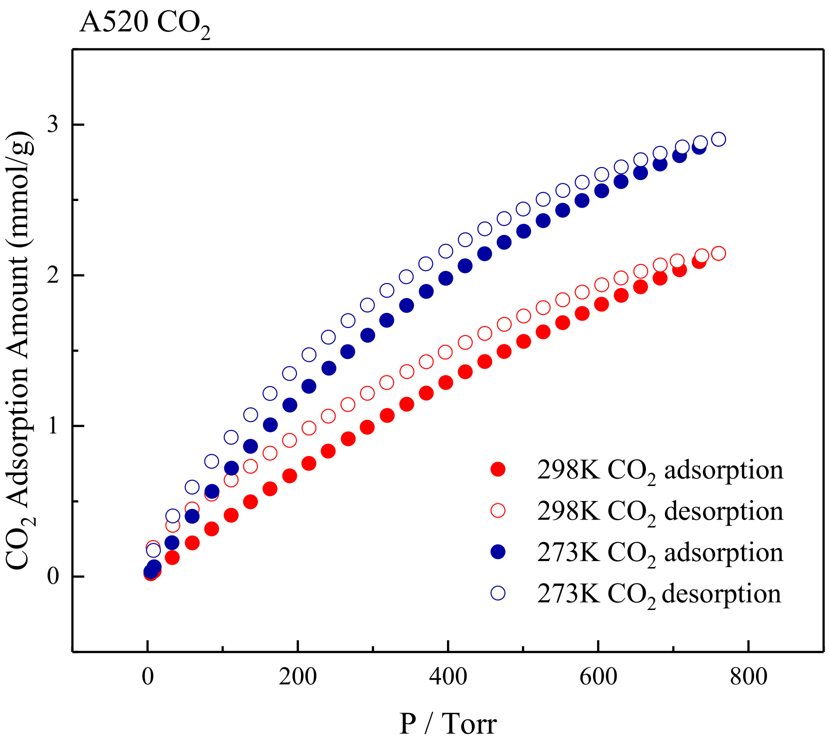

Figure S16. A520 CO_2 isotherm.

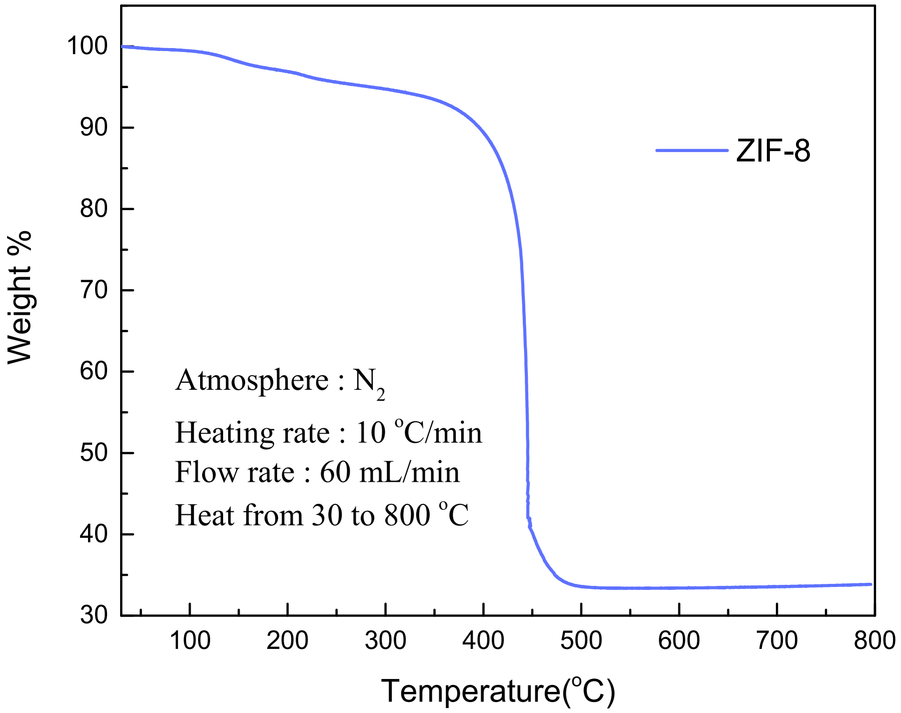

Figure S17. TGA curve of ZIF-8.

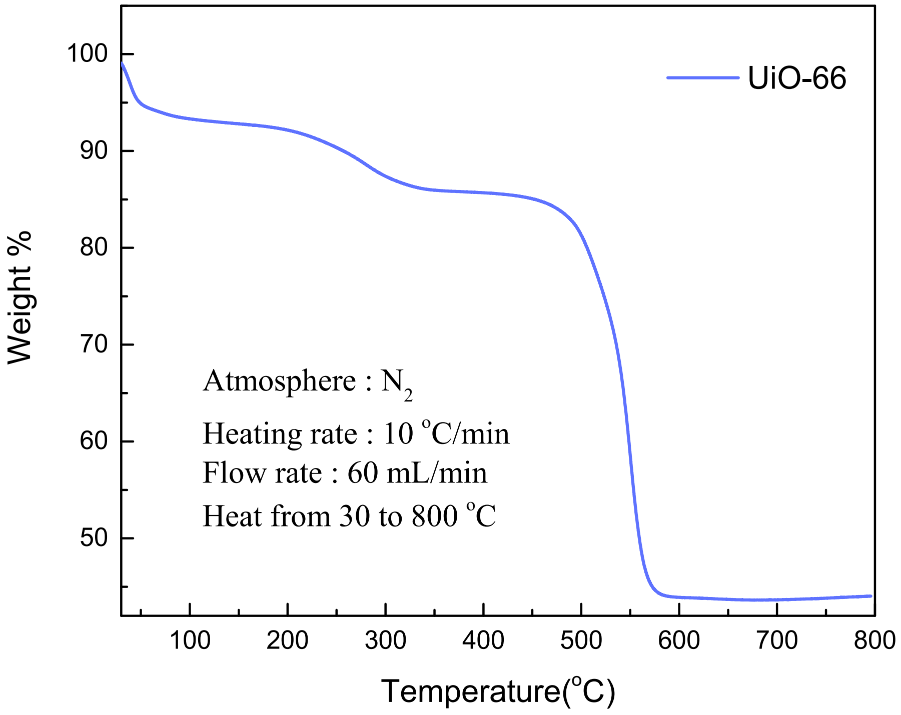

Figure S18. TGA curve of UiO-66.

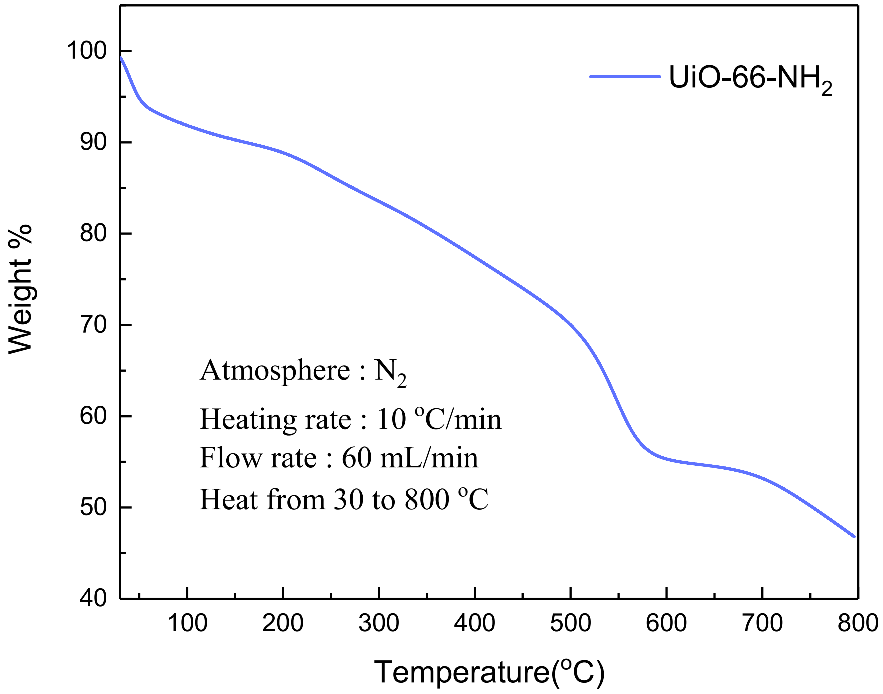

Figure S19. TGA curve of UiO-66-NH_2.

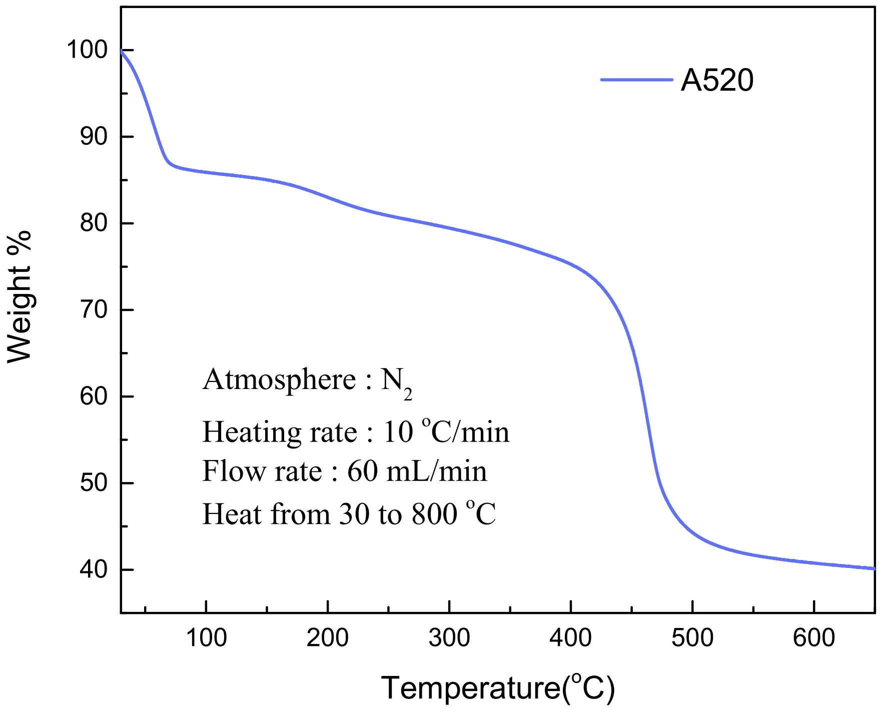

Figure S20. TGA curve of A520.

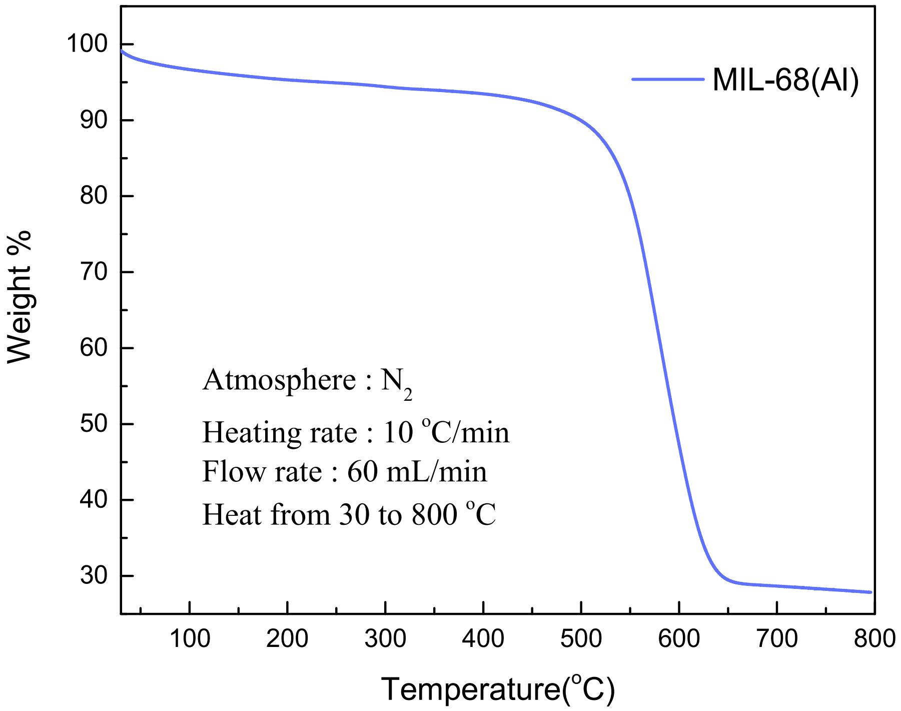

Figure S21. TGA curve of MIL-68(Al).

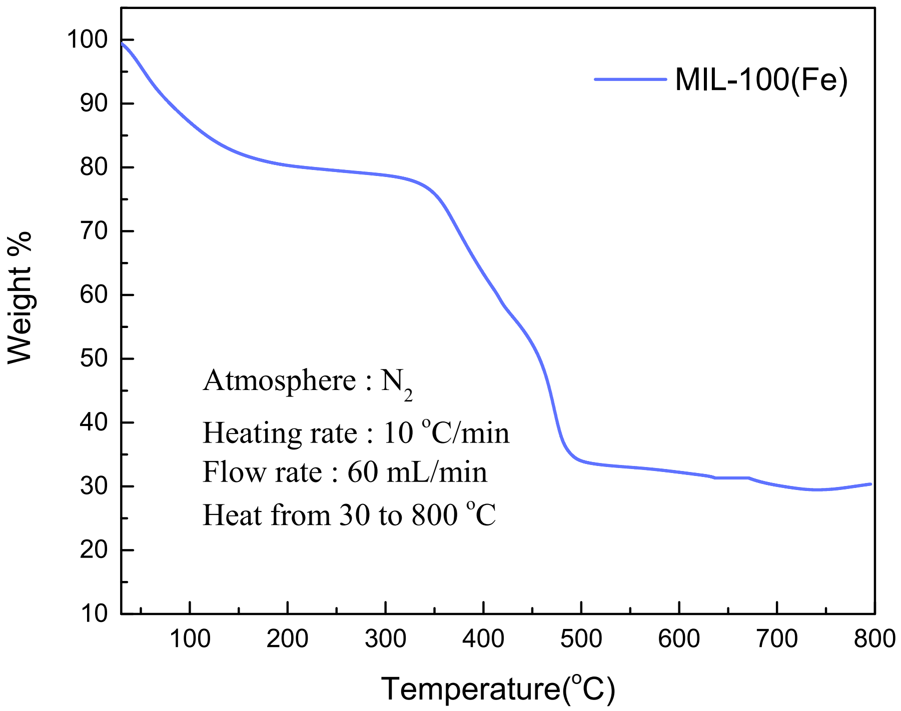

Figure S22. TGA curve of MIL-100(Fe).

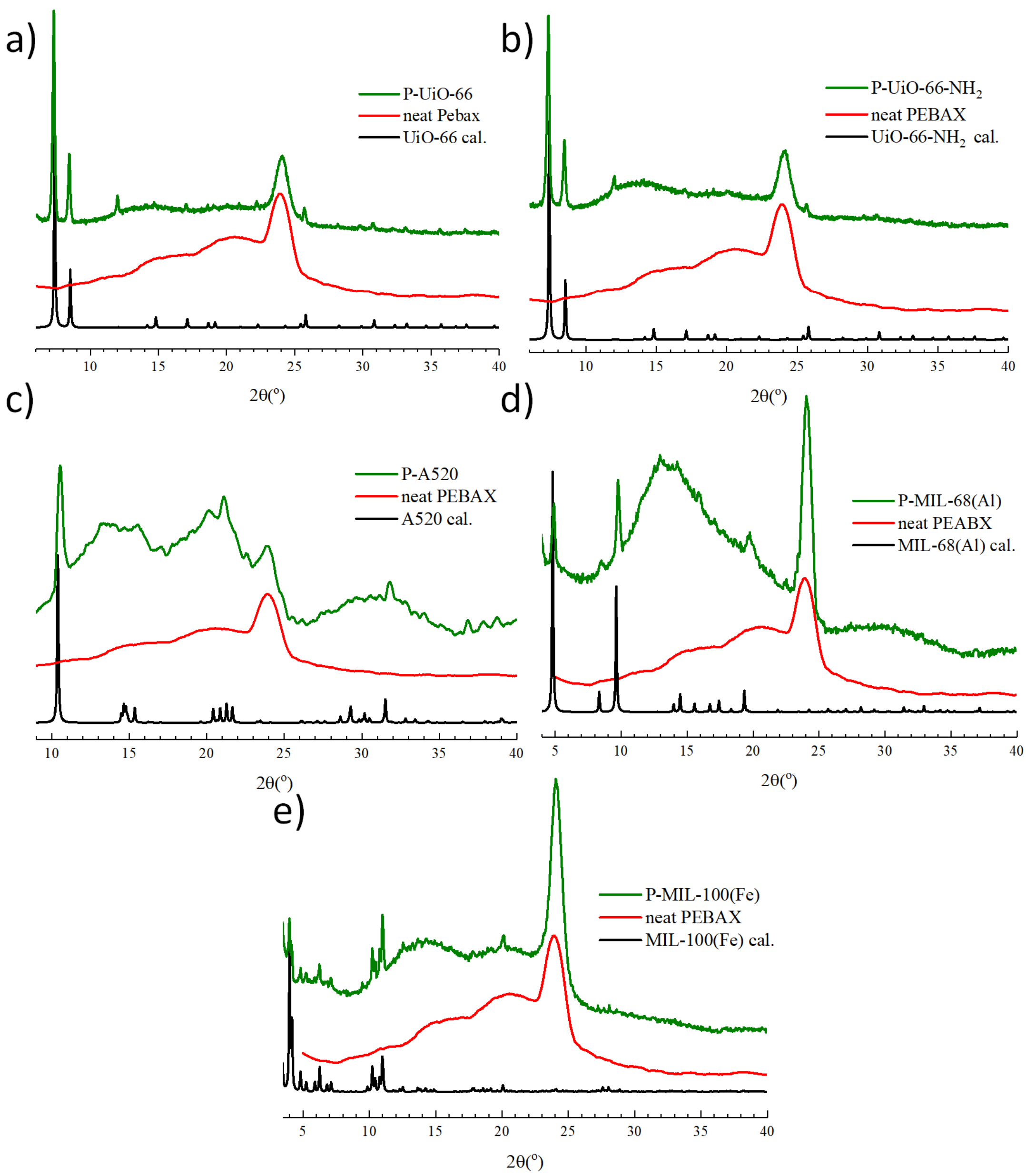

Figure S23. PXRD pattern of (a)P-UiO-66, (b)P-UiO-66-NH$_2$, (c)P-A520 (d)P-MIL-68(Al), (e)P-MIL-100(Fe).

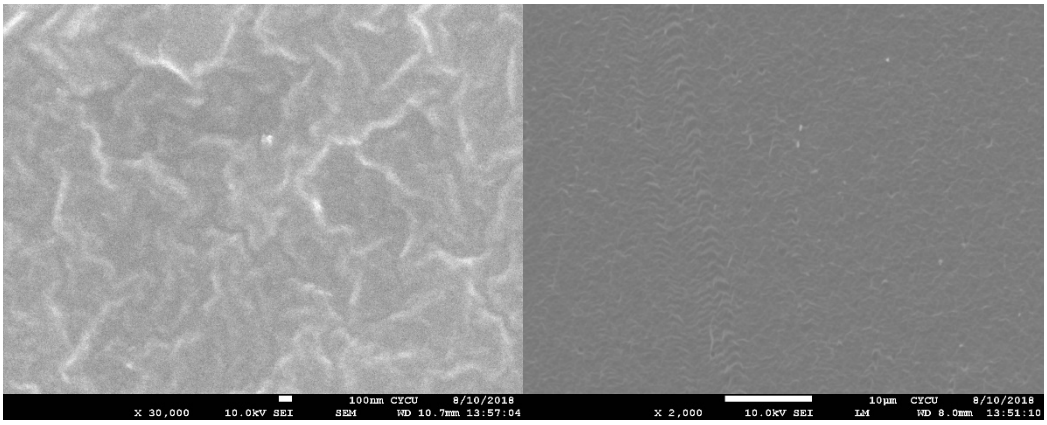

Figure S24. Neat PEBAX SEM image (left: surface, right:cross-section).

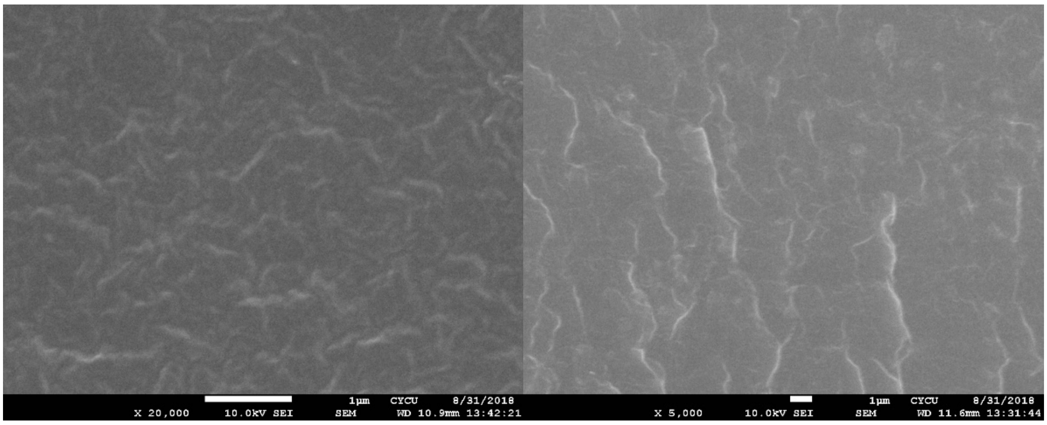

Figure S25. P-Z1 SEM image (left: surface, right:cross-section).

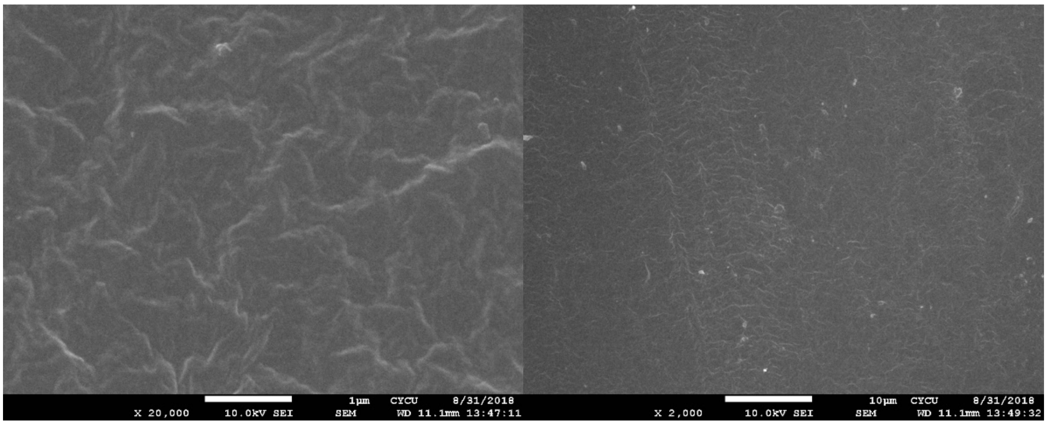

Figure S26. P-Z3 SEM image (left: surface, right:cross-section).

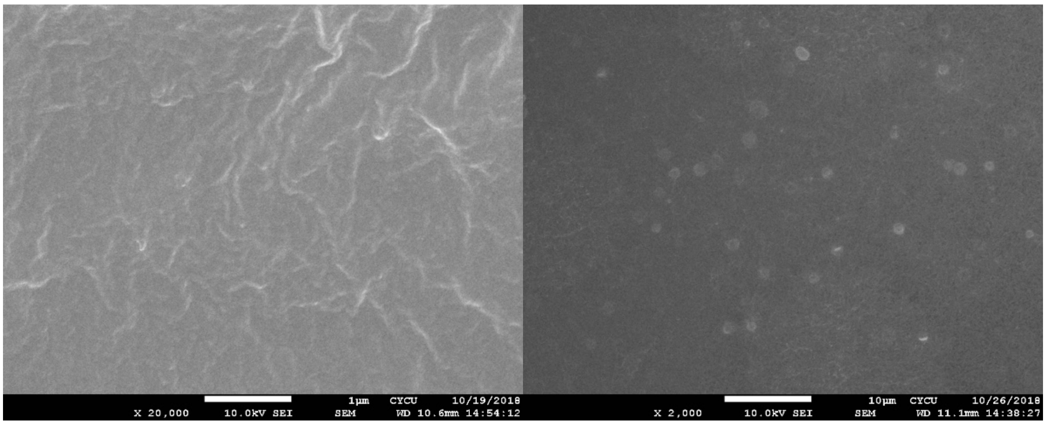

Figure S27. P-Z5 SEM image (left: surface, right:cross-section).

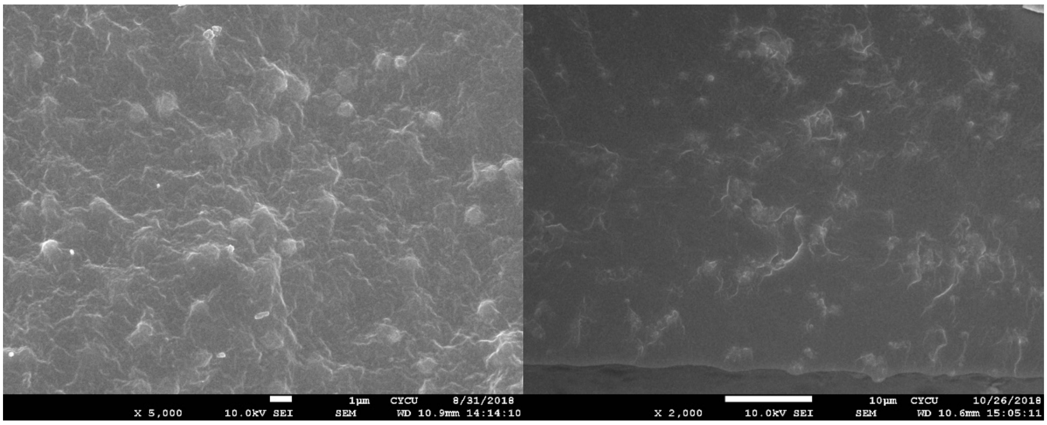

Figure S28. P-Z8 image (left: surface, right:cross-section).

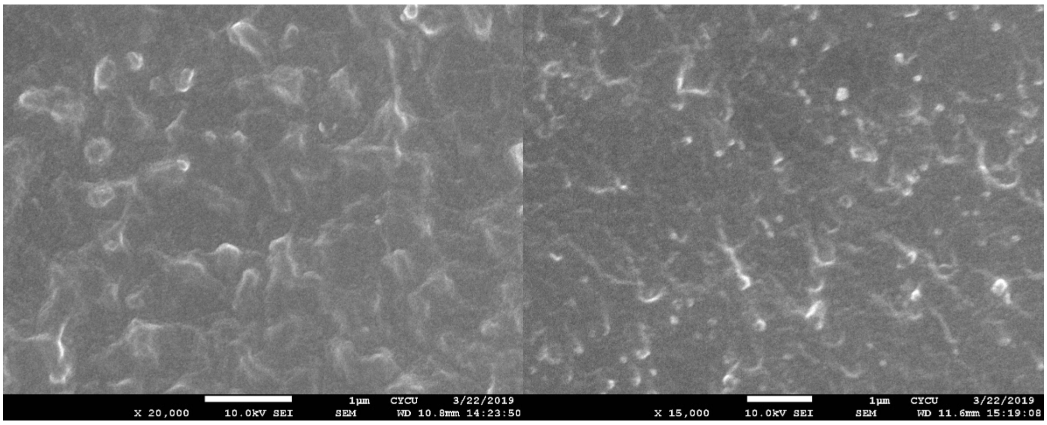

Figure 29. P-Z10 image (left: surface, right:cross-section).

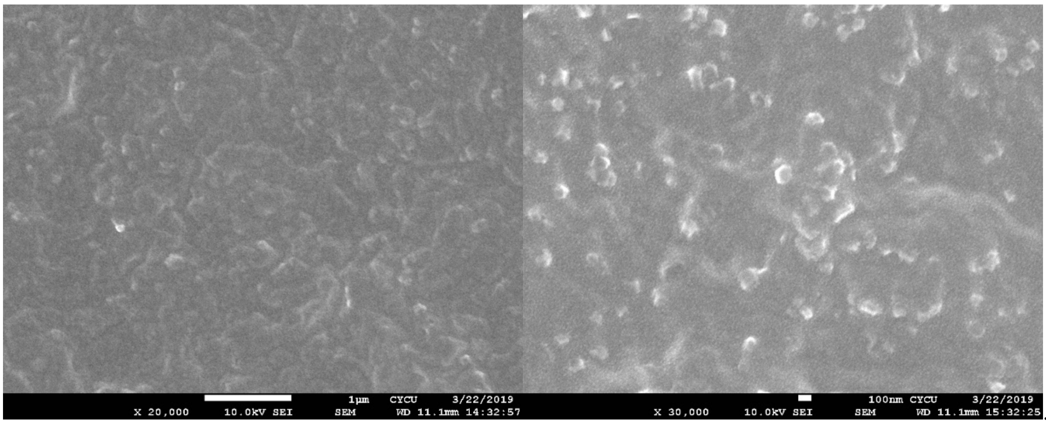

Figure S30. P-Z20 image (left: surface, right:cross-section).

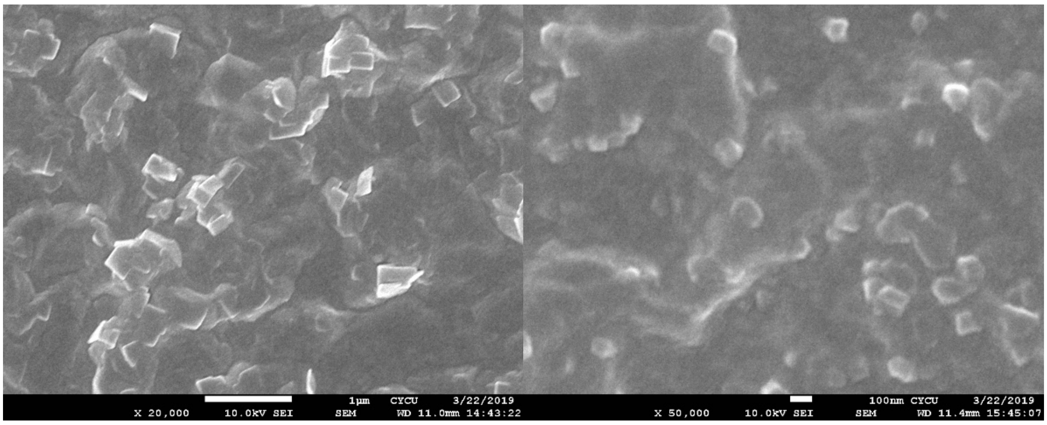

Figure S31. P-Z30 image (left: surface, right:cross-section).

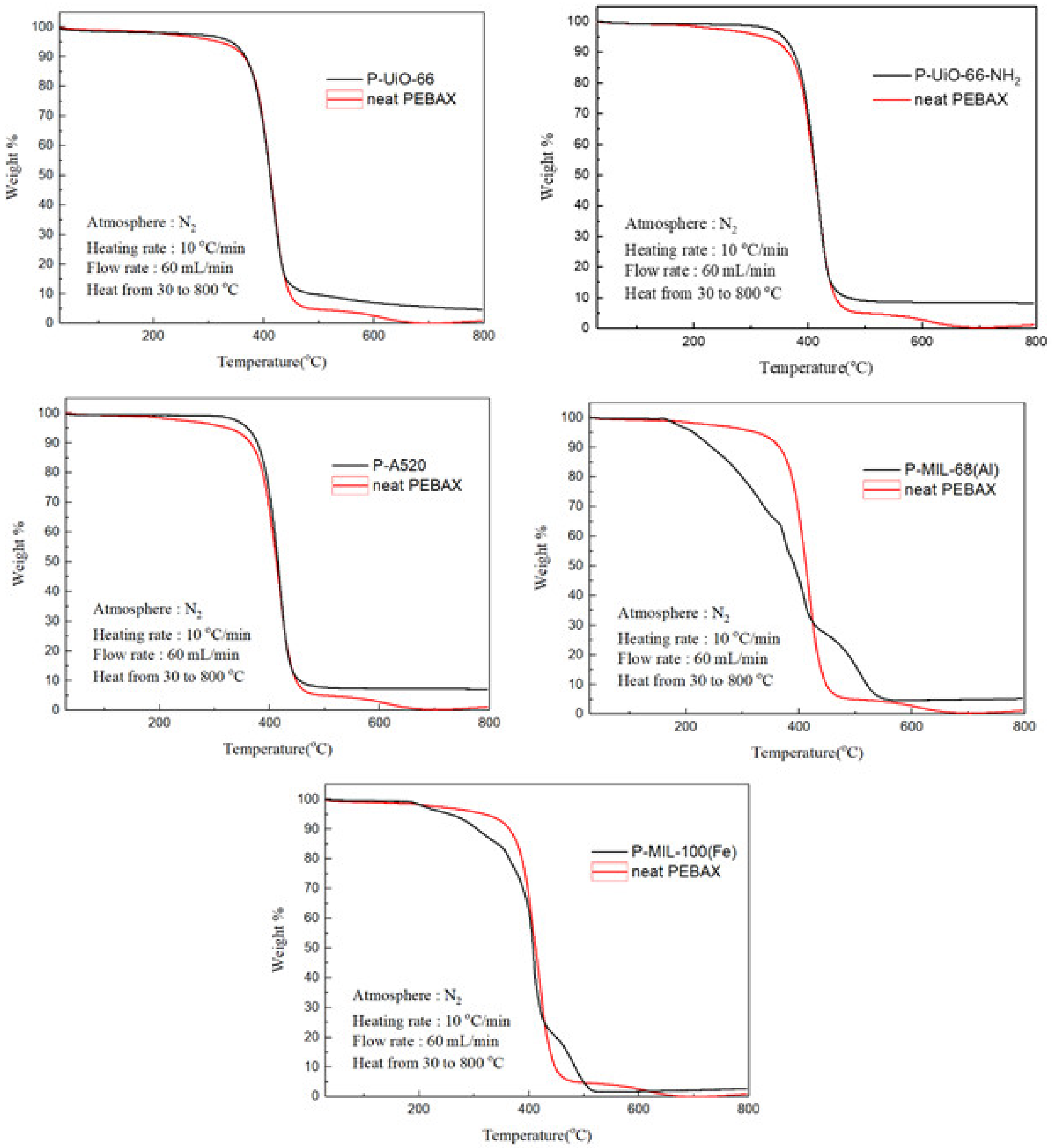

Figure S32. TGA curve of Pebax/5 wt% MOF MMMs and neat Pebax.

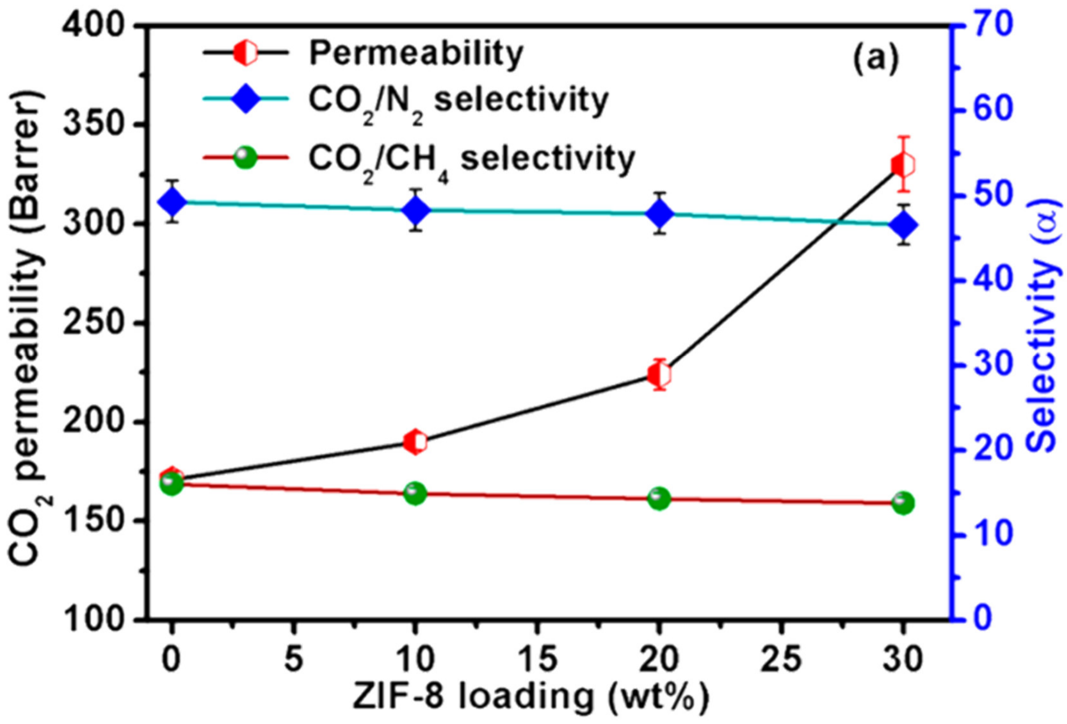

Figure S33. The gas permeability and gas separation selectivity of Pebax/ZIF-8 MMMs in the literature.

Table S1 N_2 Adsorption

	Adsorption (cm^3/g STP)	Surface Area (m^2/g)	Total Pore Volume (Å)	Pore Size (cm^3/g)
ZIF-8	432.87	2013.45	0.70	8-15
UiO-66	390.69	1420.78	0.68	8-12
UiO-66-NH_2	303.19	1155.83	0.45	15-20
MIL-53(Al)	350.54	1412.07	0.53	11-13
A520	144.89	542.21	0.44	8-18
MIL-68(Al)	371.93	1630.98	1.55	10-17
MIL-100(Fe)	413.09	1093	0.71	18-24

Table S2 CO_2 Adsorption

	CO_2 Adsorption at 298 K (mmol/g)	CO_2 Adsorption at 273 K (mmol/g)
ZIF-8	0.9	1.6
UiO-66	3.2	4.1
UiO-66-NH_2	3.5	5.3
MIL-53(Al)	1.5	1.8
A520	2.2	2.9

MIL-68(Al)	2.6	4.2
MIL-100(Fe)	1.4	2.5

Table S3. PEBAX/n wt% ZIF-8 MMMs gas separation efficiency

Item	P_{CO2} (Barrer)	P_{N2} (Barrer)	Selectivity
neat PEBAX	166.30	3.44	48.25
P-Z1	171.30	3.46	49.41
P-Z3	196.00	3.93	49.79
P-Z5	195.16	3.80	51.32
P-Z8	194.49	3.76	51.61
P-Z10	199.57	3.70	53.88
P-Z20	229.05	4.10	55.80
P-Z30	263.94	4.65	56.75

Table S4. PEBAX/5wt% MOF GPA measurement

Item	P_{CO_2} (barrer)	P_{N_2} (barrer)	Selectivity
**P-UiO-66**	**189.77**	**2.20**	**85.94**
**P-UiO-66-NH_2**	**183.96**	**3.61**	**50.89**
**P-A520**	**161.39**	**2.79**	**57.75**
**P-MIL-68(Al)**	**183.55**	**2.34**	**78.16**
**PMIL-100(Fe)**	**192.15**	**2.77**	**69.24**
